# Community‐Level Metabolic Shifts Following Land Use Change in the Amazon Rainforest Identified by a Supervised Machine Leaning Approach

**DOI:** 10.1111/1758-2229.70088

**Published:** 2025-04-23

**Authors:** Md Abdul Wadud Khan, Brendan J. M. Bohannan, Kyle M. Meyer, Ann M. Womack, Klaus Nüsslein, James P. Grover, Jorge L. Mazza Rodrigues

**Affiliations:** ^1^ Department of Biology The University of Texas Arlington Texas USA; ^2^ Institute of Ecology and Evolution University of Oregon Eugene Oregon USA; ^3^ Department of Microbiology University of Massachusetts Amherst Massachusetts USA; ^4^ Department of Land, Air and Water Resources University of California Davis California USA; ^5^ Environmental Genomics and Systems Biology Division Lawrence Berkeley National Laboratory Berkeley California USA

**Keywords:** carbon and nitrogen cycles, forests, pastures, shotgun metagenomics, soil virome

## Abstract

The Amazon rainforest has been subjected to high rates of deforestation, mostly for pasturelands, over the last few decades. This change in plant cover is known to alter the soil microbiome and the functions it mediates, but the genomic changes underlying this response are still unresolved. In this study, we used a combination of deep shotgun metagenomics complemented by a supervised machine learning approach to compare the metabolic strategies of tropical soil microbial communities in pristine forests and long‐term established pastures in the Amazon. Machine learning‐derived metagenome analysis indicated that microbial community structures (bacteria, archaea and viruses) and the composition of protein‐coding genes were distinct in each plant cover type environment. Forest and pasture soils had different genomic diversities for the above three taxonomic groups, characterised by their protein‐coding genes. These differences in metagenome profiles in soils under forests and pastures suggest that metabolic strategies related to carbohydrate and energy metabolisms were altered at community level. Changes were also consistent with known modifications to the C and N cycles caused by long‐term shifts in aboveground vegetation and were also associated with several soil physicochemical properties known to change with land use, such as the C/N ratio, soil temperature and exchangeable acidity. In addition, our analysis reveals that these alterations in land use can also result in changes to the composition and diversity of the soil DNA virome. Collectively, our study indicates that soil microbial communities shift their overall metabolic strategies, driven by genomic alterations observed in pristine forests and long‐term established pastures with implications for the C and N cycles.

## Graphical abstract

A machine learning (ML)‐derived metagenomic analysis of soils collected from Amazon forests and long‐term established pastures showed different microbial community strategies towards carbohydrate metabolism and energy allocation. The ML approach allowed the development of metabolic maps that explained process‐level modifications to the C and N biogeochemical cycles. The study also provided the first glimpse of the Amazon rainforest DNA virome impacted by deforestation.

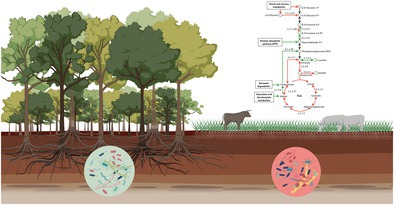

## 1 Introduction

The Amazon is the largest continuous rainforest on Earth and provides essential ecosystem services at regional and global scales. It harbours the largest collection of plants and animal species in the world (Dirzo and Raven 2003) and balances the flux of atmospheric gases (Betts et al. 2008). Despite these known benefits, forest clearing has proceeded at an alarming rate over the last few decades (Danielson and Rodrigues 2022). The process of deforestation and the subsequent land use change concurrently alters the biological and geochemical compositions of the soil (Neill et al. 1997; Fearnside 1999; Feeley and Silman 2009; Rodrigues et al. 2013), which are known to have important consequences for ecosystem‐scale processes (Durrier et al. 2021; Obregon et al. 2022).

Ecologists have long studied the contribution of plant and animal communities to ecosystem functions within tropical forests (Eva et al. 2004; Feigl et al. 2006; Foley et al. 2007) and the impact of deforestation on these communities (Fearnside 1999; Feeley and Silman 2009). In contrast, microbial ecologists have started to explore similar sets of questions only in the last 15 years, but have already demonstrated that ecosystem conversion alters bacterial (Jesus et al. 2009; Khan et al. 2019; Mirza et al. 2020), archaeal (Taketani and Tsai 2010; Navarrete et al. 2011; Meyer et al. 2020) and fungal (Mueller et al. 2016) community composition in the Amazon. Such studies, however, largely relied on single gene markers (e.g., regions of a ribosomal or a protein‐coding gene) to infer the taxonomic composition of microbial communities. More recently, metagenomic approaches have been used to examine the impacts of land‐use change on soil microbiomes in the Amazon basin (Obregon et al. 2022; Meyer et al. 2017; Kroeger et al. 2020; Venturini et al. 2022; Goss‐Souza et al. 2017; Pedrinho et al. 2019, 2020), but these studies either addressed questions associated with a specific microbial functional group (such as methanogens or methanotrophs) or a specific biogeochemical process (such as methanogenesis and methanotrophy). In addition, viruses remain neglected in Amazon soils even though they are the most abundant biological entity in soils, with an estimated 10^8^–10^10^ viral particles per gramme of soil (Jansson 2023). Viruses not only serve as the largest reservoir of genetic material (Weinbauer and Rassoulzadegan 2004), but also may play important roles in controlling microbial populations and the biogeochemical processes they mediate (Trubl et al. 2018; Emerson 2019; Starr et al. 2019). Although a few studies have been conducted on soil viromes using metagenomic information, none of them were in the context of land use type in the Amazon rainforest.

To examine the effects of aboveground differences on microbiomes derived from two of the most common land use types in the Amazon, pristine forests and pastures, we combined deep metagenomic sequencing and a machine learning (ML) strategy. ML models are powerful statistical approaches to study complex multivariate relationships that might not be linear in nature (Goodswen et al. 2021; Walsh et al. 2023), for example, organismal interactions, relative gene abundances, and biological activity. There are numerous resources for ML, but the approach scarcely used information‐rich metagenomic datasets (McElhinney et al. 2022). Here, we applied a supervised ML architecture for its predictive features, as opposed to unsupervised, or inherently exploratory in nature. First, classification was performed on a subset of samples, and the training set was used for ML model construction. Second, the model was used for categorising the remaining metagenomes. Finally, a leave‐one‐out cross validation procedure was performed to estimate model performance. This supervised ML strategy allowed us to: (i) estimate the composition of soil microbiomes and protein‐coding genes and draw distinct metabolic relationships between microbiomes under primary forests versus established pastures, (ii) identify differential abundances of key protein‐coding genes involved in ecologically relevant biochemical processes and (iii) examine the Amazon soil DNA virome to gain a comprehensive understanding of its alterations in response to two different plant covers.

## 2 Materials and Methods

### 2.1 Site Description and Sampling

The study was performed at the Amazon Rainforest Microbial Observatory (ARMO), located at Fazenda Nova Vida in the State of Rondônia, Brazil (10°10′18.71″ S, 62°47′15.67″ W) (Rodrigues et al. 2013). This location has one of the highest rates of deforestation in the Brazilian Amazon (Mapbiomas Amazonía 2022), representing the last four decades of man‐made ecosystem alterations occurring in this region. Detailed information about the vegetation and soil properties of our study sites has been described elsewhere (Neill et al. 1997; Feigl et al. 2006). Primary forests are typical wet ‘Terra Firme’ forests with the majority of the trees yet to be identified, while pastures were established and have been in continuous use since 1972 after a slash‐and‐burn procedure followed by aerial seeding of the fast‐growing grasses *Urochloa brizantha* and *Panicum maximum*. The sampling strategy and sample processing were described in detail elsewhere (Rodrigues et al. 2013; Meyer et al. 2017). For this study, five forest soil samples and five long‐term established pasture soil samples were collected with a 10‐cm depth by 5‐cm diameter corer after the removal of the litter layer. Soil cores were collected in 2010, frozen on the spot, transported on dry ice to the laboratory, and stored at −80°C until DNA extractions were performed.

Sampling plots are 3.66 km apart and are a well‐documented observational chronosequence (Walker et al. 2010; Chazdon 2013) of long‐term forest‐to‐pasture conversion (Neill et al. 1997; de Moraes et al. 1996; Steudler et al. 1996). In addition to the close geographical distances, these experimental plots were selected for the following reasons: (1) plots have identical soil genesis, drainage, slope and climate conditions, limiting any concerns of spatial variability, (2) the consequences of land use change in this observational chronosequence are supported by previous studies that span 30 years, providing a historical context for our study (Rodrigues et al. 2013; Durrier et al. 2021; Feigl et al. 2006; Khan et al. 2019; Mirza et al. 2020, 2014; Navarrete et al. 2011; Meyer et al. 2020, 2017; Mueller et al. 2016; Kroeger et al. 2020; de Moraes et al. 1996; Steudler et al. 1996; Cenciani et al. 2009; Neill et al. 1995; Hamaoui et al. 2016; Melillo et al. 2001; Garcia‐Montiel et al. 2001) and (3) the opportunity of performing the largest metagenomic sequencing of individual samples of Amazonian soils to date.

### 2.2 Soil Physicochemical Analysis

At the time of sampling, in situ measurements of soil pH and temperature were taken following standard protocols. Soil samples were taken for measurement of the following physicochemical properties: total organic matter (OM), total carbon (C), total nitrogen (N), phosphorus (P), potassium (K^+^), sulphur (S), calcium (Ca^+2^), magnesium (Mg^+^), aluminium (Al^+3^), exchangeable acidity (H^+^ + Al^+3^), base saturation (V), sum of bases (SB), cation exchange capacity (CEC), aluminium saturation index (m), iron (Fe), manganese (Mn), zinc (Zn), copper (Cu), boron (B) and carbon/nitrogen (C/N) ratio. All analyses were performed at the Laboratório de Fertilidade do Solo, Department of Soil Sciences, University of São Paulo, Brazil.

### 2.3 Soil DNA Extraction and Sequencing

Soil DNA extraction was performed with the PowerLyzer Powersoil DNA Isolation Kit (Mobio Inc., Carlsbad, CA). Five DNA extractions were performed per soil sample and pooled for metagenomic library preparation with the TrueSeq DNA kit (Illumina Inc., San Diego, CA). Sequencing was performed on the Illumina HiSeq platform to generate 6.4 billion paired‐end reads with an average insert size of 150 bp.

### 2.4 Sequence Processing and Annotation of Metagenomes

Quality filtering of the raw sequences was performed using the default criteria as set by the Rapid Annotation using Subsystems Technology for Metagenomes (MG‐RAST) pipeline (i.e., trimming of low‐quality bases, removal of artificial replicate sequences and filtering of sequences with greater than 5 ambiguous bases). As part of the MG‐RAST pipeline version 3.2, paired‐end reads were joined using the fastq‐join algorithm. Single‐end reads with 150 bp length that could not be joined were retained. Quality‐filtered sequence reads were searched against the Kyoto Encyclopedia of Genes and Genomes (KEGG) (Kanehisa and Goto 2000) and RefSeq (Pruitt et al. 2007) databases for functional and taxonomic (bacterial, archaea and viruses) annotations, respectively. The annotations were conducted at an *e*‐value cutoff of 1e−5, a minimum identity cutoff of 60%, and a minimum alignment length cutoff of 15 (default parameters) for the annotations of metagenome sequences. Following the annotations, the datasets were exported in biom file format and merged by QIIME 1.8.0 (Caporaso et al. 2010) for the downstream analyses.

The Amazon soil metagenomic dataset is publicly available and deposited in the MG‐RAST database (Marguran 2005) as well as in the Joint Genome Institute IMG/M database (Meyer et al. 2008) under the following accession numbers: 1080879 to 1080888.

### 2.5 Soil Community Structure Assessment

Rarefaction plots were generated to test whether sampling effort was sufficient. Next, we compared the relative abundances of major bacterial, archaeal and viral taxa and functional categories of genes (KEGG level 2) between forest and pasture ecosystems. Alpha diversities were calculated using species (or gene) richness, defined as the total number of unique species (or genes) present in a defined area, and the Shannon index (*H′*), a nonparametric value that takes into consideration the proportion of species (or genes) in a particular dataset (Chen et al. 2021). Bray–Curtis dissimilarity, a numerical value of the species (or gene) composition between two different sites (Chen et al. 2021), was used to calculate the pairwise dissimilarities and perform principal coordinate analysis (PCoA) between metagenomes. To test the hypothesis that forests and pastures have differential metagenome profiles, PCoA plots were generated using the species‐level abundance dataset of bacteria and archaea, where functional categories at KEGG level 2 were fitted as vectors. Here, we used the two *R* packages—‘*ape*’ (Paradis et al. 2004) and ‘*vegan*’ (Dixon 2003) to calculate the association of each ecosystem with different functional attributes of the soil bacterial and archaeal communities. Fitting of soil variables onto PCoA plots was performed with the vegan package of the R software, and the significance of correlations was assessed with 999 random permutations.

### 2.6 Supervised ML

We employed the supervised ML classifier Random Forest, using the R package ‘*randomForest*’ (Liaw and Wiener 2002), to classify a subset of microbial species and protein‐coding genes (explanatory variables) that were discriminatory between land uses (response variable). First, we estimated the accuracy of the ML model by selecting a subset of the samples that was randomly partitioned as a training set to build the model. Next, the trained dataset was used to classify the remaining samples as a test set.

Next, we performed a validation procedure to calculate the average estimated generalisation error of the ML classifier set and compared values against the baseline error for random guessing. The leave‐one‐out cross validation is a statistical method that uses all samples for model training but leaves one sample out (Goodswen et al. 2021). In our study, the left‐out sample was then classified and results were compared to those obtained for the training dataset. The procedure repeated itself for all samples. We calculated the ratio (*k*) of baseline error and estimated error and considered a ratio of two or higher (*k* ≥ 2) for an acceptable classification, meaning the Random Forest ML classifier performed at least twofold better than random guessing.

Finally, we conducted an importance score calculation for each feature classified in the training dataset (i.e., species or protein‐coding gene) by determining the increase in estimated error when a feature was ignored. In our analysis, we set a very conservative alpha level of 0.0001 as the cutoff for importance scores.

### 2.7 Statistical Analysis

To determine whether differences in the relative abundances of major taxonomic and functional categories between pristine forests and long‐term established pastures were statistically significant, the Mann–Whitney test with 1000 permutations was used. Performing the same statistical test, we compared the alpha diversities and pairwise taxonomic distances between samples. Analyses of similarities (ANOSIM) tests were performed to assess whether metagenome compositions were significantly different across the terrestrial ecosystems.

To identify microbial and viral species, and protein‐coding genes at KEGG level 4 (KEGG orthology [KO]) that were significantly different across forests and pastures, we evaluated our results using the DESeq2 tool (Love et al. 2014) to calculate fold‐difference in gene abundance, which was represented as the log_2_‐fold‐change (log_2_FC). A score of zero indicated that the KO gene or species had statistically the same proportional abundance in metagenomes of both land use types. A positive value for a given gene or species indicated overrepresentation in forest metagenomes compared to pastures, while a negative value indicated the reverse pattern. The DESeq2 tool, a negative binomial Wald test, is particularly useful for metagenomic datasets, because observations have high variation among biological replications, and sample variance is typically greater than the sample mean (Love et al. 2014).

To increase the robustness of identifying abundant species and genes between land uses and their importance for soil ecosystems and prevent false positives in our dataset, we combined results from both the Random Forest ML model and DESeq2. The algorithms QIIME 1.9.0 and GraphPad Prism 7.00 were used for performing statistical analyses and visualising the results.

## 3 Results

### 3.1 Soil Metagenome Profiles

We obtained a mean of about 636 million (± 12%) reads per sample, totalling 6.4 billion paired‐end reads with an average length of 171 bp. Following quality filtering of metagenomes through MG‐RAST (version 3.3.6), the sequence reads were mapped to the KEGG (Vol. 36) and RefSeq (Vol. 37) databases, where each read was annotated according to the KO groups and species (bacteria, archaea and viruses) associated with the identified alignments. A vast majority of the sequence reads (83.7%) passed the quality control by MG‐RAST (Table S1) and were submitted for assignment: 21.7% of the sequences were assigned to predicted proteins with known function, 47.9% assigned to predicted proteins of unknown function, 0.5% assigned to rRNA genes and the remaining 13.5% failed to be assigned to any category. Sequencing reads varying from 60,373,709 to 76,838,373 were annotated as KOs with 783,394–1,143,853 taxonomically identified as belonging to bacteria, 20,661–51,138 reads as archaea and 18,307–32,903 as viral reads. Our analysis obtained a total number of unique 5607 KO genes, with 23,949 being of bacterial origin, 706 archaeal and 1774 viral. Before downstream analyses, all samples were rarefied to their lowest sequencing depth to bring them to equal values to remove sequencing effort bias. The rarefaction plots showed that the diversity levels of these metagenomic datasets tended to saturation and were suitable for a comprehensive analysis of the microbial communities and their metabolic potentials in soils under both land use types (Figure S1).

### 3.2 Bacterial and Archaeal Community Differences Between Forests and Pastures

We observed substantial variation of major bacterial and archaeal taxa between forest and pasture soils. With over 26% (±0.82%; standard error of mean [SEM]) in forests and 19% (±0.22%; SEM) in pastures (*p* < 0.001), *Alphaproteobacteria* was the dominant soil bacterial class under both plant ecosystems and showed the largest variation in response to land use (Figure S2A). Other major bacterial groups that differed significantly between forests and pastures (*p* < 0.001) included: *Actinobacteria* and *Firmicutes*, which increased from 13.1% (±0.21%; SEM) to 16.65% (±0.41%; SEM) and 11.39% (±0.52%; SEM) to 14.4% (±0.17%; SEM), respectively. *Euryarchaeota* and *Thaumarchaeota*, were the two most differentially abundant phyla (*p* < 0.001) within the Domain *Archaea*, where the former increased from 39.3% (±0.59%; SEM) to 55.48% (±0.52%; SEM), and latter decreased from 27.41% (±0.47%; SEM) to 7.73% (±0.72%; SEM) in relative proportions, between forests and pastures, respectively (Figure S2B). We also identified differentially abundant bacterial (Table S2A) and archaeal (Table S2B) species in each land use. Using both criteria set by DESeq2 and Random Forest methods in this study, we identified 131 bacterial species that were enriched in forest soils, and 189 species that were enriched in pasture soils. *Bradyrhizobium* was the most dominant bacterial genus in the forest soils, whereas *Bacillus* and *Arthrobacter* dominated pasture soils. With the same criteria, 101 archaeal species were estimated to be differentially abundant in forests, with an unclassified crenarchaeotal genus the most dominant. On the other hand, pasture soils had 51 species that were differentially abundant with an unclassified euryarchaeal genus the most dominant.

### 3.3 Diversity Patterns of Taxonomic Lineages and Protein‐Coding Genes

**FIGURE 1:**
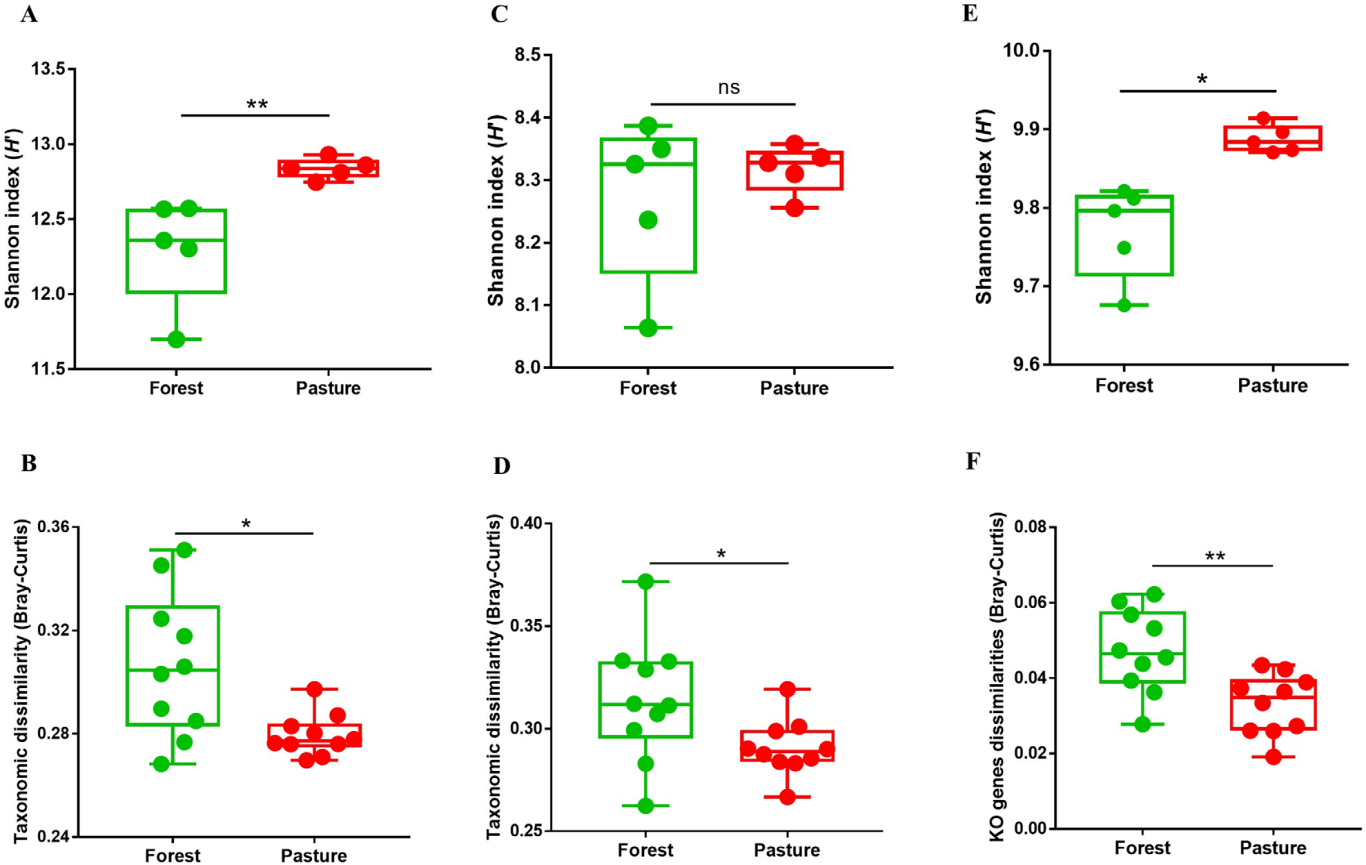
Metagenomic‐derived response of bacterial (A, B), archaeal (C, D) and protein‐coding genes (E, F) to forest‐to‐pasture conversion. Shannon index and Bray–Curtis similarity were used for estimating alpha (*n* = 5) and beta diversities (*n* = 10 for pairwise similarity), respectively, where mean values of diversity were depicted. Error bars represent standard error of the mean (SEM). Statistical significance was calculated using Mann–Whitney test with 1000 permutations. Symbols * and ** indicate significance values of *p* < 0.05 and *p* < 0.01, respectively.

**FIGURE 2:**
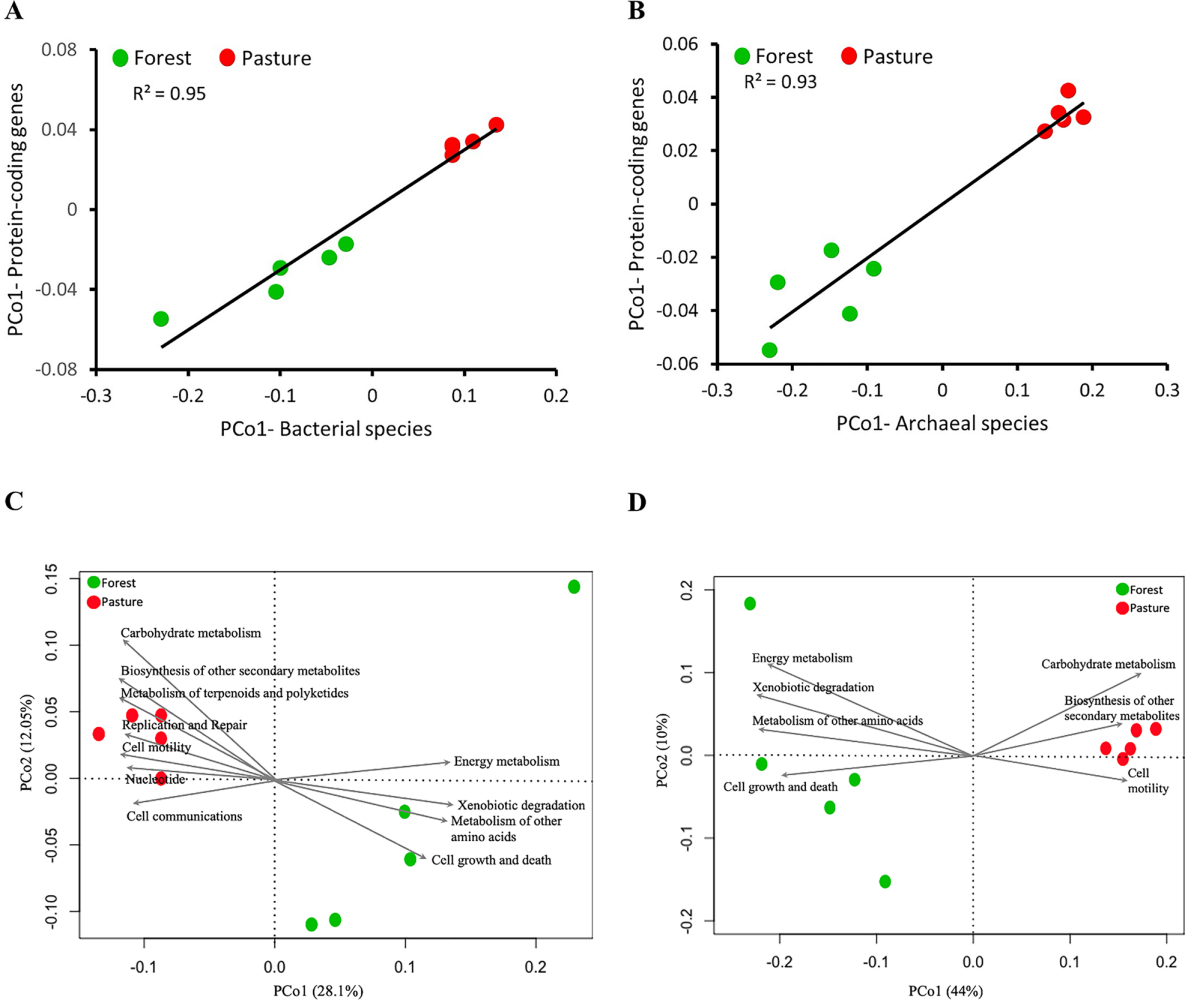
Relationships of protein‐coding gene compositions with bacterial and archaeal species compositions across forest and pasture metagenomes. The relationships of compositional similarities are visualised using the principal coordinates loadings for the first axes (PCo1). Protein‐coding genes demonstrated significantly positive correlations with bacterial (A) and archaeal (B) community compositions. Vector fitter principal coordinates analysis showed that bacterial (C) and archaeal (D) communities have distinct clustering patterns by soil types, where vectors represent the association of the functional characteristics at KEGG level 2. The vectors are shown with arrows pointing to the direction of an increase in the gradient for the corresponding variable, and with length proportional to the correlation between ordination and variable. The circles (forests, green; pastures, red) represent the relative positions of each soil taxonomic community. The significances (*p* values) of the vectors were calculated based on 999 random permutations of the data. Here, only functional characteristics that were estimated to be significantly (*p* < 0.01) associated with PCo1 are shown. Bray–Curtis metric was used for estimating distances between different samples.

Pastures had increased alpha (*p* < 0.01; Figure 1A) but decreased beta (*p* < 0.05; Figure 1B) diversities of bacterial communities in comparison to forests. Archaeal diversity exhibited a slightly different pattern, with no significant difference in alpha diversity (Figure 1C) and significantly lower beta diversity in pastures relative to forests (*p* < 0.05; Figure 1D). Diversities of KO genes resembled those of bacteria, with an increase in alpha and a decrease in beta in pasture soils (alpha: *p* < 0.05; Figure 1E and beta: *p* < 0.01; Figure 1F). We then asked whether the bacteria and archaea diversity results co‐varied with protein‐coding gene diversities. We selected the principal coordinate loadings of the first axis (PCo1), which explained 28.1% and 44% of variation in the metagenome compositions for bacteria and archaea, respectively. Our data showed strong compositional similarities of KO gene profiles with bacterial (*R* ^2^ = 0.95, *p* < 0.05; Figure 2A) and archaeal (*R* ^2^ = 0.93, *p* < 0.05; Figure 2B) species profiles.

### 3.4 Impact of Land Use Change on Major Soil Functional Characteristics

The ordination plots based on PCoA showed that the taxonomic composition in the forests clustered apart from that in pastures (ANOSIM: *R* = 0.74, *p* = 0.007 and *R* = 0.99, *p* = 0.006, respectively; Figure 2C,D). Next, we fit KEGG level 2 functions as vectors onto the ordination, and several functional attributes were significantly associated with the bacterial PCo1 (28.1%) across different soil samples (Figure 2C). Positive correlations of PCo1 with energy metabolism (*r* ^2^ = 0.92, *p* < 0.001), cell growth and death (*r* ^2^ = 0.98, *p* < 0.001), xenobiotics biodegradation and metabolism (*r* ^2^ = 0.96, *p* < 0.001) and metabolism of other amino acids (*r* ^2^ = 0.96, *p* < 0.01), as indicated by the direction of the arrows, demonstrated that increasing abundance of genes associated with these functions was strongly associated with forest samples, whereas pasture samples were located at opposite ends of the ordination. Similarly, pasture soils had an increasing presence of sequences associated with carbohydrate metabolism (*r* ^2^ = 0.86, *p* < 0.01), cell motility (*r* ^2^ = 0.89, *p* < 0.001), metabolism of terpenoids and polyketides (*r* ^2^ = 0.58, *p* < 0.05), nucleotide (*r* ^2^ = 0.67, *p* < 0.05), cell communications (*r* ^2^ = 0.71, *p* < 0.05) and biosynthesis of other secondary metabolites (*r* ^2^ = 0.91, *p* < 0.01), whereas forest soils were at the decreasing spectrum of the ordination. The protein‐coding genes for the same cellular functions were in the opposite direction when archaeal taxa were considered (Figure 2D). Overall, pastures had significantly higher relative abundances for protein‐coding genes for the following functional categories: carbohydrate metabolism, replication and repair, nucleotide metabolism, metabolism of terpenoids and polyketides and cell motility, in comparison to forests (Figure S3).

### 3.5 Differential Abundance of Protein‐Coding Genes and Metabolic Pathways

**FIGURE 3:**
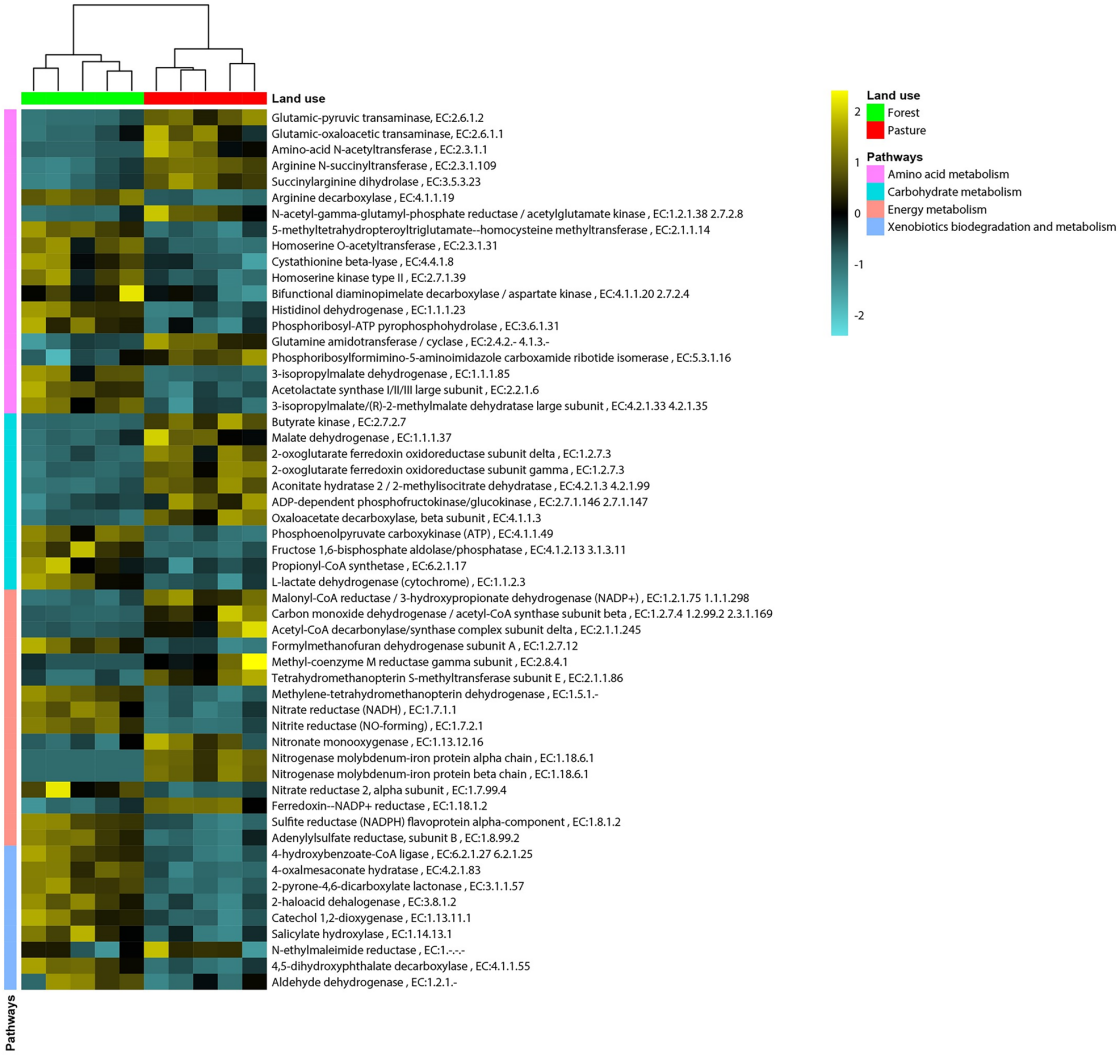
Heat map comparison of *Z*‐scores calculated from protein‐coding gene abundances between forest and pasture metagenomes. Only subset of genes encoding KEGG ECs involved in nutrient cycling is shown (see Table S2 for a complete list of all discriminatory protein‐coding genes). Only genes that passed selection criteria for both DESeq2 (BH‐adjusted *p* < 0.05) and Random Forest (importance score > 0.0001) methods were considered.

Next, we focused on determining which metabolic pathways (KEGG level 3) and associated protein‐coding genes (KEGG level 4) were associated with the differences observed in major functional categories (KEGG level 2) between forest and pasture soils. Our differential abundance analysis identified 935 KO genes showing large differences between forest and pasture metagenomes, of which 541 belong to KEGG Enzyme Commission (EC) categories. A complete list of KO genes with log_2_FC values and the *p* values of Wald tests is provided in Table S3. Our analysis showed that the major differences of forest and pasture soil metagenomes were associated with genes involved in carbohydrate, energy, and amino acid metabolisms and xenobiotic degradation, which are functionally connected to other pathways (Figure 3).

#### 3.5.1 Carbohydrate Metabolism

**FIGURE 4:**
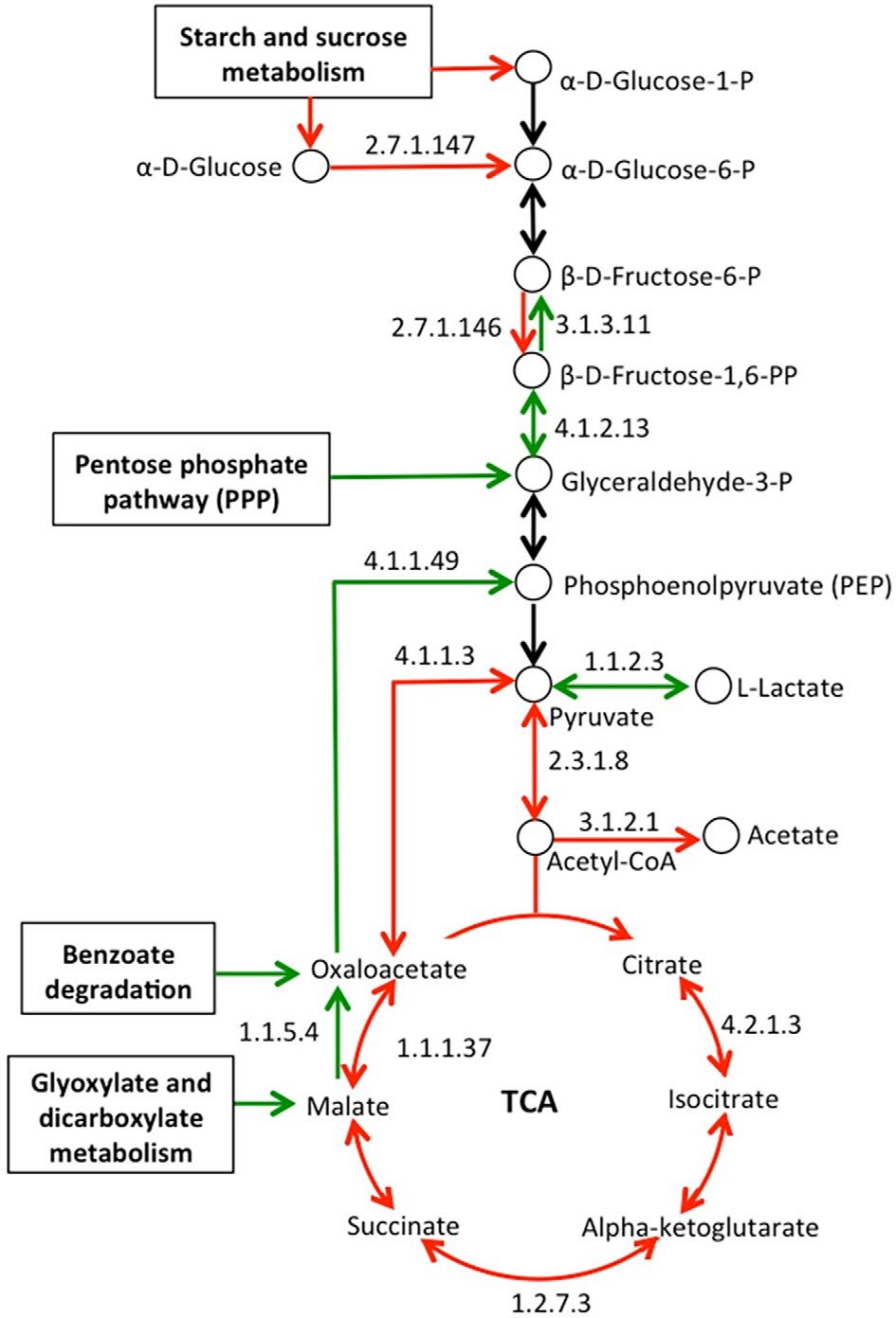
Metabolic map for glycolysis/gluconeogenesis and the tricarboxylic acid (TCA) cycle based on differentially abundant genes observed in forest and pasture metagenomes. Only genes that passed selection criteria for both DESeq2 (BH‐adjusted *p* < 0.05) and Random Forest (importance score > 0.0001) methods are represented. Arrows indicate the enzyme‐mediated steps of the pathway, with KEGG EC numeric classification representing the reaction being catalysed. A green arrow means that the abundance of a gene encoding a KEGG EC enzyme was higher in the forest, while red means higher in the pasture. Black arrows indicate genes with similar abundances between forest and pasture metagenomes.

Pastures were estimated to contain elevated numbers of KEGG EC genes associated with the metabolism of starch, sucrose and galactose (Table S3). In addition, KO genes involved in the transport of mono‐ and di‐saccharides were far more abundant in pastures, of which log_2_FC values ranged from −0.39 to −3.01. Unlike pasture metagenomes, which had elevated counts of glycolysis‐specific genes (e.g., ADP‐dependent phosphofructokinase/glucokinase [EC 2.7.1.146, EC 2.7.1.147]), forest metagenomes had higher counts of genes specific for gluconeogenesis (e.g., fructose 1,6‐bisphosphate phosphatase [EC 3.1.3.11]; Figure 4). In addition, genes associated with the pentose phosphate pathway (PPP) and the conversion of oxaloacetate to phosphoenolpyruvate (PEP; phosphoenolpyruvate carboxykinase [EC 4.1.1.49]) had higher relative abundances in the forest metagenome samples. Furthermore, we observed considerable variation between forest and pasture metagenomes in the frequencies of genes that are involved in central metabolic pathways. For example, genes associated with the tricarboxylic acid (TCA) cycle were estimated to be higher in pasture soils (Figure 4). In addition, pastures had higher abundances of genes affiliated with the fermentation of acetate (e.g., acetyl‐CoA hydrolase [EC 3.1.2.1]) and butanoate (e.g., butyrate kinase [EC 2.7.2.7]), whereas genes associated with the fermentation of propanoate (propionyl‐CoA synthetase [EC 6.2.1.17]) and lactate (l‐lactate dehydrogenase, cytochrome [EC 1.1.2.3]) were elevated in forests.

#### 3.5.2 Energy Metabolism

**FIGURE 5:**
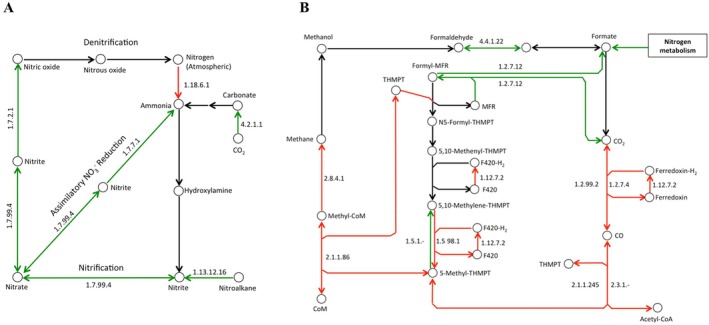
Metabolic maps for the nitrogen (A) and methane (B) metabolisms based on differentially abundant genes observed soil metagenomes under forests and pastures. Only genes that passed selection criteria for both DESeq2 (BH‐adjusted *p* < 0.05) and Random Forest (importance score > 0.0001) methods are represented. Arrows indicate the enzyme‐mediated steps of the pathway, with a KEGG EC numeric classification representing the reaction being catalysed. A green arrow means that the abundance of a gene encoding a KEGG EC enzyme was higher in the forests, while red means higher in the pastures. Black arrows indicate genes with similar abundances between forest and pasture metagenomes.

The most prominent differences observed were related to the genes associated with N and methane (CH_4_) metabolisms (Figure 5A). Forest metagenomes had an overrepresentation of EC genes involved in nitrification (nitronate monooxygenase [EC 1.13.12.16], nitrate reductase 2 alpha subunit [EC 1.7.99.4]), denitrification (nitrite reductase, NO‐forming [EC 1.7.2.1]) and assimilatory nitrate reduction (nitrate reductase, NADH [EC 1.7.7.1]) (Figure 5A). Conversely, the estimated representation of genes involved in biological N_2_ fixation was higher in the pasture metagenomes (nitrogenase molybdenum‐iron protein alpha, and beta chains [EC 1.18.6.1] with log_2_FC values of −5.76 and − 5.92, respectively). In CH_4_ metabolism, several genes involved in methanogenesis were enriched in pasture soil metagenomes (e.g., carbon monoxide dehydrogenase [EC 1.2.7.4], acetyl‐CoA decarbonylase/synthase complex subunit delta [EC 2.1.1.245], tetrahydromethanopterin S‐methyltransferase [EC 2.1.1.86] and methyl‐coenzyme M reductase gamma subunit [EC 2.8.4.1]). Other genes involved in methanotrophy were enriched in the forest soil samples (methylene‐tetrahydromethanopterin dehydrogenase [EC 1.5.1.‐], formylmethanofuran dehydrogenase subunit A [EC:1.2.7.12]) (Figure 5B; Table S3).

#### 3.5.3 Amino Acid Metabolism

The overall abundance of genes involved in amino acid metabolism was not statistically different between forests and pastures. However, we observed overrepresentation of certain genes in forest metagenomes, especially those encoding for enzymes involved in key steps of oxaloacetate‐derivative amino acids such as methionine, threonine (Figure S4A), PPP‐derivative amino acids including histidine (Figure S4B) and pyruvate‐derivative branched‐chain amino acids (valine, leucine and isoleucine) (Figure S4C). In addition, degradation genes for branched‐chain amino acids were also higher in forest metagenomes (Table S3). As a contrast, pasture metagenomes were associated with the metabolism of alpha‐ketoglutarate, another TCA cycle intermediate (Figure 4). In addition, there was increased gene abundance related to the biosynthesis of arginine from glutamate and proline from arginine (Figure S4D).

#### 3.5.4 Xenobiotics Biodegradation and Metabolism

We determined higher gene sequence counts (*p* < 0.05) in forest metagenomes associated with the degradation of diverse aromatic compounds including benzoate, chlorocyclohexane and chlorobenzene, dioxin, nitrotoluene and polycyclic aromatic hydrocarbons (Figure S5). These pathways seem to be linked and contribute to the production of pyruvate and oxaloacetate that might act to support the TCA cycle and replenish its intermediates, a mechanism known as anaplerosis, which is used in the biosynthesis of essential compounds such as amino acids.

#### 3.5.5 Other Metabolic Processes

Pasture soils had a higher abundance of several genes affiliated with chemotaxis, flagellar assembly and regulation of the actin cytoskeleton (Table S3). In addition, we estimated several differentially abundant genes between forest and pasture soils that were associated with plant–microbe and microbe–microbe interactions. For example, genes associated with the biosynthesis of other secondary metabolites such as benzoxazinoid, which are plant secondary metabolites typically from grass species, and a beta‐lactam resistance gene observed to be higher in pasture metagenomes. In contrast, a streptomycin synthesis gene encoding for a streptomycin‐6‐phosphatase (EC 3.1.3.39) was higher in forest metagenomes. Within the two‐component system pathway, the abundance of genes for multidrug transport proteins (K07788, K07789) was greater in forest soils, while bacitracin resistance response regulator proteins (K11629, K11630) were estimated to be higher in pasture metagenomes. Several genes (K13448, K13429, K13462, K13428, K13447 and K13424) that are known to be associated with plant–pathogen interaction pathways were more abundant in pasture metagenomes, while none were detected in forest soil metagenomes.

### 3.6 Relationship Between the KO Gene Composition and Soil Physicochemical Properties

To identify potential drivers of community genomic composition, we performed an environmental fitting of seven soil variables that were significantly correlated with protein‐coding genes at the community level onto an ordination plot via PCoA. KO genes clustered by land use type (ANOSIM: *R* = 0.98, *p* = 0.007; Figure S6). Negative correlations of PCo1 with C/N ratio (*r* ^2^ = 0.95, *p* < 0.001), temperature (*r* ^2^ = 0.97, *p* < 0.001), base saturation (V; *r* ^2^ = 0.78, *p* < 0.01) and iron (*r* ^2^ = 0.72, *p* < 0.01) demonstrated that pasture soil samples have stronger associations with these factors, whilst forest soil samples are at opposite ends of these physicochemical properties (Table S5). Similarly, in forest soil samples, PCo1 is correlated with increasing exchangeable acidity (H^+^ + Al^3+^), sulphur and boron, whereas pasture samples were associated with decreases in these soil physicochemical properties.

### 3.7 Impact of Land Use Change on Viral Composition and Diversity

**FIGURE 6:**
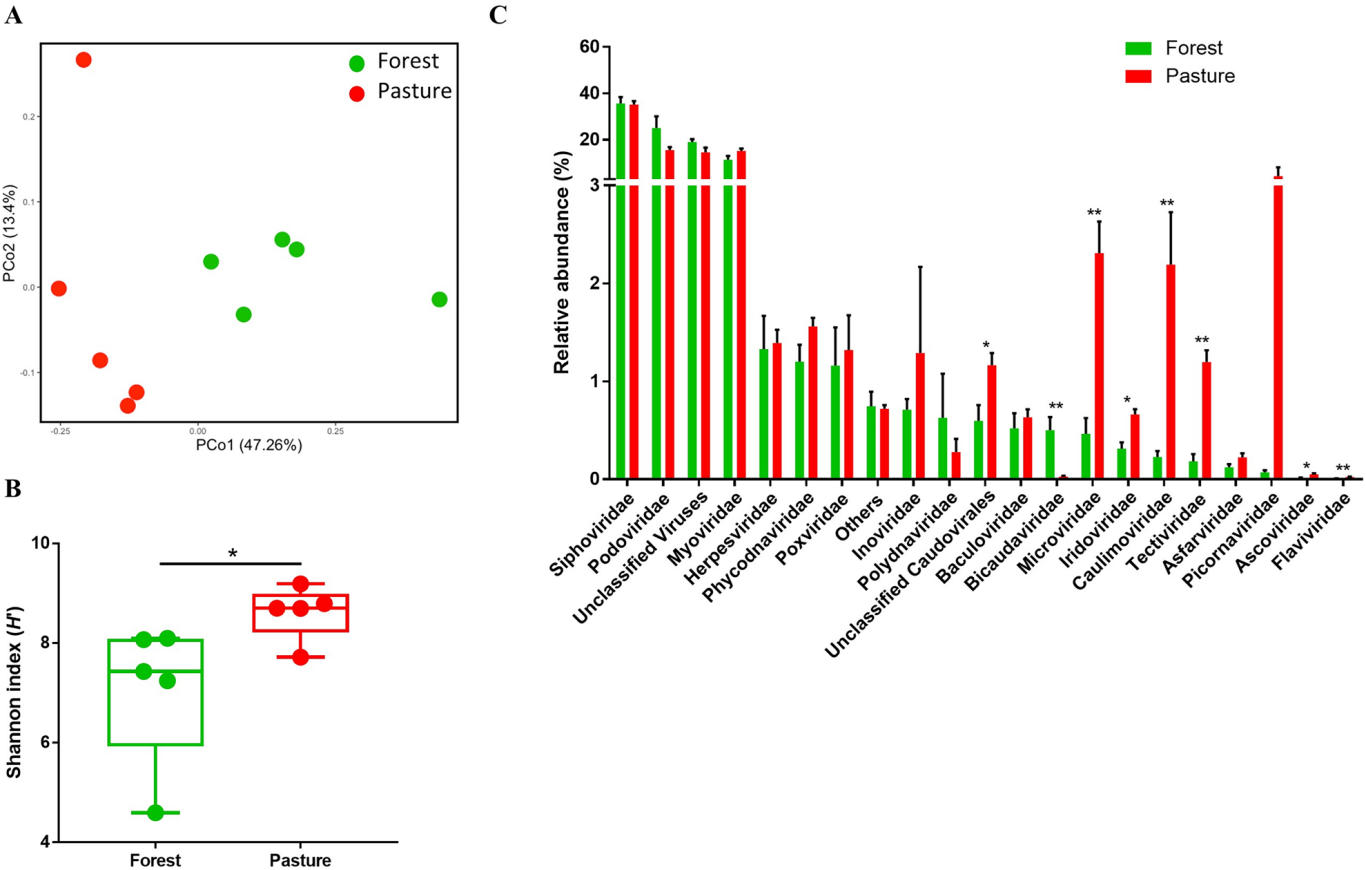
Virome community structure in the metagenomes obtained from soils under the Amazon forest (green) and pasture (red). (A) Ordination plot from principal coordinate analysis showed that forest viral composition is distinct from that of pastures. (B) Alpha diversity as estimated by Shannon index is significantly higher in pastures compared to forests. (C) Relative abundances of viral communities are estimated at family level in forest and pasture metagenomes. Error bars represent standard error of the mean (SEM). Symbols * and ** indicate significance values of *p* < 0.05 and *p* < 0.01, respectively, which were calculated using a Mann–Whitney test with 1000 permutations.

While evaluating the impact of land use change on genomic content of microbial communities, we observed a large number of reads associated with viral sequences and asked whether land use change impacted also the soil DNA virome. We observed that alpha diversity of the soil virome was higher in pasture soils compared to forest soils (Figure 6A), with significant compositional differences (ANOSIM: *R* = 0.67, *p* = 0.008; Figure 6B). We compared the viral populations at the family level between the two land use types and estimated that *Siphoviridae*, *Podoviridae*, an unclassified family, and *Myoviridae* were the most abundant groups, collectively comprising over 80% and 90% of the sequences found in pasture and forest soils, respectively (Figure 6C). While the relative abundance of sequences for the above families did not differ between land uses, we observed significant differences for several viral families, such as *Bicaudaviridae*, *Microviridae*, *Caulimoviridae*, *Tectiviridae* and *Flaviviridae*. We identified 25 viral species that were differentially enriched in forests and 67 species that were differentially enriched in the pastures (Table S4). In addition, we observed significant compositional associations of viral taxa with bacterial (*R* ^2^ = 0.84, *p* < 0.05) and archaeal taxa (*R* ^2^ = 0.93, *p* < 0.05), as well as KO genes (*R* ^2^ = 0.86, *p* < 0.05) (Figure S7A–C).

## 4 Discussion

By applying a strategy of deep sequencing (mean 636 million reads per sample) and a supervised ML approach, we identified differences in microbiome composition, gene content and inferred metabolism in soils of two land use types in the Amazon basin: pristine forests and pastures that were created 38 years ago after deforestation. In addition, we observed differences in virome diversity and composition between forest and pasture soils. Soil viromes are understudied, especially in the Amazon, owing to the limited number of soil samples collected in tropical ecosystems. With the newly discovered importance of viruses to soil biogeochemical cycles (Jansson 2023; Weinbauer and Rassoulzadegan 2004), this work provides a glimpse of their diversity.

The alteration of bacterial and archaeal compositions and diversities determined in pristine forests and long‐term established pastures observed in this study mirrors previous observations made with the use of single gene markers such as the 16S rRNA gene (Rodrigues et al. 2013; Khan et al. 2019; Navarrete et al. 2011), confirming the utility of Random Forest as a supervised ML classifier for metagenomic analysis. While other Amazon metagenomic studies have yielded 2.9–13.5 million reads per sample (Goss‐Souza et al. 2017; Pedrinho et al. 2019), this study obtained 636 million reads per sample. With the advent of new sequencing technologies, the number of metagenomic studies will continue to increase exponentially, and ML approaches, supervised or unsupervised, will be needed to handle large molecular datasets.

The microbial community compositions of both forests and pastures were associated with protein‐coding (KO) genes (Figure 1A,B), suggesting a direct relationship between species presence, their associated KO genes and metabolic processes (Figure 1C,D). More importantly, our study demonstrates that a distinct difference in plant cover between forests and pastures results in increased homogenisation of protein‐coding genes (KO; Figure 1F), which may be explained by the reduced substrate heterogeneity in pastures, which are dominated by only two plant species (both grasses). These shifts in biochemical pathways suggest changes to the microbial metabolic strategies related to energy allocation and nutrient acquisition. For example, significant metagenomic differences were noticed between forests and pastures for genes associated with energy and carbohydrate metabolisms; two processes of importance to C and N cycling. While most of the major functional characteristics are maintained in both land use systems, relative abundances of many specific genes in these two processes substantially differed, indicating the potential for differences in physiological status and strategies related to nutrient absorption. Recently, Gralka et al. (2023) demonstrated that distinct preferences for glycolytic or gluconeogenic carbon substrates are predicted by genomic contents of 186 heterotrophic bacteria, and how this dichotomy of carbon sources could relate to differences in C and N utilisation. While these findings were observed for marine strains, it is likely that the same principle of metabolic partitioning is at play in soils, where there is a staggering variety of carbon resources.

Plants are major contributors of organic carbon to soils through rhizodeposition (Zhalnina et al. 2018), and microbial communities utilise an abundance of carbon compounds for growth and maintenance (Grandy and Neff 2008). Accordingly, our forest and pasture metagenomes show distinct genomic signatures for resource utilisation. Pasture soils are known to be rich in cellulosic and hemicellulosic litter from grasses, and one of the two grass species that dominates our sampled pasture is *U. brizantha*, which stores nutrients including starch in its rhizomes (Deinum et al. 1996). Along with animal dung in pastures, these carbon compounds potentially represent rich sources of easily degradable carbohydrates, as evidenced by increased values of permanganate oxidisable carbon, an indicator of soil organic matter lability, previously assessed for soil samples obtained from the same pastures (Durrier et al. 2021). The increased occurrence of genes involved in starch and sucrose metabolism, and the transport of mono‐ and di‐saccharides suggests that microbes may rely more on exogenous saccharides than other complex substrates. There is evidence that this is happening in our study systems. Pasture metagenomes have elevated counts of genes for glycolysis, TCA cycle and acetate fermentation in comparison to forest metagenomes that were enriched in genes involved in gluconeogenesis (Figure 4). Higher abundance of PPP genes in forest metagenomes may provide flexibility to glycolysis/gluconeogenesis by funnelling glyceraldehyde‐3‐P, an intermediate of the PPP, into these pathways. Our results also suggest that forest metagenomes had higher biosynthetic capacity of essential amino acids of oxaloacetate, pyruvate and pentoses, like ribose‐5‐phosphate, which can serve as a precursor for nucleotide syntheses (White 2007). On the other hand, higher gene abundances for amino acid biosynthesis in the α‐ketoglutarate family were observed in pastures. An amino acid of this family, glutamate (Figure S4D), is the precursor for chlorophyll synthesis (Barton 2005), which may imply an increased capacity for photosynthesis and carbon fixation in pastures. This agrees with our previous findings of increased β‐glucosidase activity (Durrier et al. 2021) and microbial biomass (Cenciani et al. 2009) for the same pastures evaluated in our study.

In previous work, we reported the molecular composition of soil organic matter across land uses and observed significant and pronounced presence of aromatic (C═C) and ketone/quinone/amide (─C═O/─C(═O)─/─C(═O)N─) groups in forest sites (Durrier et al. 2021). Along with the low concentration of C substrates, there is also a higher diversity of C compounds due to the complex secondary chemistry of a myriad of forest vegetation (Ohno et al. 2014). These complex compounds are degraded by soil microorganisms into a few central aromatic intermediates, such as benzoate, catechol and protocatechuate (Figure S5), through peripheral catabolic pathways that are subsequently channelled into the β‐ketoadipate pathway by *ortho* ring cleavage (Fuchs et al. 2011). Among others, nitrate‐respiring bacteria, dominated by pseudomonads, cleave the benzene‐ring of these aromatic compounds (Fuchs et al. 2011; Heinaru et al. 2001), which leads to the generation of TCA cycle intermediates. Our current observations are consistent with this past work; for example, we observed that *Pseudomonas* spp. and genes related to the degradation of aromatic compounds are only enriched in forest soils (Figure S5), suggesting the maintenance of the TCA cycle by replacing oxaloacetate and pyruvate that are removed for the biosynthesis of glucose and amino acids. The overrepresentation of genes associated with glyoxylate metabolism in forest soil samples may also contribute to carbon anaplerosis by supplying malate and its subsequent conversion to oxaloacetate (Figure 4). Together, the carbohydrate metabolic processes in forest microbial communities not only would maintain a better trade‐off between glycolysis and gluconeogenesis, but also provide metabolic plasticity for energy harvest and anaplerotic reactions to occur, strategies that may be critical for the oligotrophic forest soil environment.

Prominent differences imposed by ecosystem conversion in the Amazon are long known to result in biogeochemical alterations to the N and C cycles (Walsh et al. 2023; Neill et al. 1995), but it required approximately 20 years to connect these differences to changes in soil microbial community structure (Obregon et al. 2022; Meyer et al. 2017; Kroeger et al. 2020; Venturini et al. 2022; Goss‐Souza et al. 2017; Pedrinho et al. 2019). With the application of a supervised ML approach to metagenomic datasets, our study is a step forward towards understanding which pathways are most impacted by ecosystem conversion, especially processes related to substrate acquisition and energy metabolism. Our data demonstrate that not only the abundance of methanogens and several known KO genes associated with methanogenesis (Figure 5B) are elevated is pastures, but also an increased capacity for glycolysis and the formation of acetate (Figure 4), with the latter being an important substrate for acetoclastic methanogenesis. In contrast, we observed that CH_4_‐consuming microbial taxa and associated genes involved in the process of methanotrophy have a lower abundance in the pasture, particularly due to the loss of proteobacterial methanotrophs including *Methylomonas*, *Methylobacterium* and *Methylocella*. Our results are in compliance with previous observations of changes in the taxonomic composition of methane‐consuming microorganisms in the same study area (Obregon et al. 2022) as well as distant pastures (1241 km apart) in Amazonia (Venturini et al. 2022).

Our supervised ML approach also highlighted differences in pathways involved in the cycling of N. The Random Forest model results indicated that forest soils have a higher gene content in association with the oxidation of nitroalkanes, suggesting that these functional moieties may serve as sources of C, N and energy for nitrifying microorganisms. For example, our results showed that KO genes associated with members of the nitrifying archaeal phylum *Thaumarchaeota* are more frequently found in forest soils. This finding agrees with our previously published results using the functional marker genes, *amo*A and *amo*B, which encode the α subunit of the ammonia monooxygenase enzyme for *Archaea* and *Bacteria*, respectively (Hamaoui et al. 2016). In addition, we observed a higher abundance of several species of *Alphaproteobacteria* in forest soils and KO genes known to be associated with steps of the N cycle, such as *Nitrobacter* sp. LIP in nitrification, and *Rhodoplanes* sp. HMD01017 in denitrification. Higher capacity for energy‐yielding denitrification in forest soils and other oligotrophic habitats leads to the increased production of nitrous oxide and nitrogen gases (Tiedje 1988), which has been shown before for Amazonian forest soils (Melillo et al. 2001). Previously, Garcia‐Montiel et al. (2001) have shown a reduction in N_2_O emissions from old pastures in comparison to pristine forests. They attributed this result to a direct consequence of low soil N availability, perhaps resulting from fast‐growing grass species (*U. brizantha* and *P. maximum*) and continued grazing of the above plant biomass. Giving support to this line of evidence, our ML analysis identified biological N_2_ fixation as the only process within the N cycle with an overrepresentation of genes present in pastures (Figure 5A). To the extent that available N declines, evidenced by a higher C/N ratio (*p* < 0.01) observed in pasture samples, there is likely a selective pressure on soil microbes to fix atmospheric nitrogen. This is consistent with previous qPCR results showing one order of magnitude increase in the *nifH* gene abundances in soils from pastures in comparison to forests (Mirza et al. 2014).

The metabolic differences we observed between forest and pasture soil microbiomes could be due to a direct influence of plant species (rhizodeposition and litter decomposition) and/or indirect alterations of soil physicochemical properties. Forest samples had significantly lower pH, temperature, C, C/N ratio and base saturation in comparison to pasture soils. Deforestation by the process of slash‐and‐burn causes a variety of changes in physicochemical factors, such as an increase in nutrient concentrations and soil temperatures (McGrath et al. 2001; Thomaz et al. 2020) that are known to impact microbial community structure and function. For example, temperature is a key factor in regulating many terrestrial processes including soil respiration (Riach and Schlesinger 1992), nitrification (MacDonald et al. 1995), denitrification (Malhi et al. 1990) and methane emissions (Venturini et al. 2022). A meta‐analysis by Rustad et al. (2001) reported that warming in the range of 0.3°C–6.0°C increased soil respiration rates by 20% and net N mineralization rates by 46%. Temperature‐induced respiration would lead to a depletion of O_2_ in the terrestrial systems, subsequently triggering anaerobic energy‐yielding processes such as fermentation, denitrification and methanogenesis. This observation has important implications for this tropical ecosystem under man‐made alterations, such as our studied area. The mean temperature difference between our study sites (24.86°C in forest vs. 27.14°C in pasture, *p* < 0.01) is similar to what is predicted by 2050 for the entire Amazon basin (Nobre et al. 2016; de Oliveira et al. 2021). While both soil systems are sources of greenhouse gases (N_2_O, NO, CO_2_ and CH_4_), increasing temperatures have a higher potential for the generation of these greenhouse gases in both ecosystems. Therefore, future studies could help to improve our understanding of the long‐term impact of climate‐driven alterations to ecosystem processes in this understudied region of our planet.

While evaluating microbial genomic differences in soils of pristine forests and long‐term established pastures, our ML approach identified a large number of virus‐derived sequences, allowing us to obtain a glimpse of the Amazon DNA virome. Viruses are reservoirs of many protein‐coding genes of prokaryotic hosts, with the potential to influence nutrient cycling, evolution, and food webs (Walsh et al. 2023). Importantly, the enormous microbial and viral deposits in the forest soil may have implications for human health yet to be fully evaluated and understood. Prist et al. (2023) observed that Amazonian municipalities with lower levels of forest fragmentation had lower numbers of diseases and infections in comparison to deforested areas in indigenous territories. Our results showed that the *Molluscum contagiosum* virus and *Vaccinia* virus, two members of *Poxviridae*, a family of dsDNA viruses, are overrepresented in forest soils. Increased incidences of poxvirus infections in cattle and humans have been reported near the Amazon region of western Colombia (Usme‐Ciro et al. 2017) and throughout Brazil (Trindade et al. 2007), coinciding with the increased rate of deforestation in the Amazon. Our results also indicated an increased presence of sequences belonging to the *Wolbachia* phage WO in forest soils. As one of the most widespread reproductive intracellular bacteria in the biosphere (Zug and Hammerstein 2012), members of the genus *Wolbachia* influence the reproductive capacity of their hosts, including mosquitoes. Giving support to the above considerations, Burkett‐Cadena and Vittor (2018) performed a metadata analysis of 87 mosquito species and informed that vectors of human pathogens were favoured by the process of deforestation, significantly increasing the risk of malaria infections. Knowledge of the disease ecology of deforestation is still rudimentary, underscoring the need for increased surveillance of potential outbreaks near deforested regions. We acknowledge that our Amazon virome results are limited to only double‐stranded DNA viruses and restricted to the identification of sequences already available in public databases, but these findings found congruency between the composition of prokaryotic species and viral species (Figure S7A,B), indicating that deforestation has consequences to at least a portion of the Amazon virome biodiversity (Figure 6C).

## 5 Conclusions

Our strategy of performing a supervised ML approach on deep metagenomic sequencing allowed the identification of specific metabolic pathways that showed significant alterations in gene abundances in soils under deforestation. These alterations imply noteworthy community‐level metabolic shifts related to energy allocation and nutrient acquisition in pristine forests and long‐term established pastures. Our results also permitted the development of metabolic maps to explain long observed alterations to the biogeochemical cycles of C and N in soils with contrasting plant covers. Finally, we provided the first glimpse of the Amazon DNA virome derived from metagenomes and showed a potential impact of deforestation in this hitherto unknown component of the Amazon soil biodiversity.

## Author Contributions

**Md Abdul Wadud Khan:** conceptualization, investigation, writing – original draft, data curation, formal analysis, validation. **Brendan J. M. Bohannan:** funding acquisition, writing – review and editing, resources, methodology. **Kyle M. Meyer:** methodology, software, formal analysis, writing – review and editing. **Ann M. Womack:** methodology, writing – review and editing, software, formal analysis. **Klaus Nüsslein:** funding acquisition, writing – review and editing, resources. **James P. Grover:** conceptualization, project administration, supervision. **Jorge L. Mazza Rodrigues:** funding acquisition, conceptualization, project administration, supervision, resources, writing – review and editing.

## Ethics Statement

The authors have nothing to report.

## Consent

The authors have nothing to report.

## Conflicts of Interest

The authors declare no conflicts of interest.

## Supporting information


**FIGURE S1.** Rarefaction curves of bacterial (A), archaeal (B), viral (C) taxonomic and protein‐coding (KO) (D) gene distributions of soil metagenomes obtained from Amazon forests and pastures. Species richness was used as a function of sequencing depth for forest and pasture samples. Green and red lines indicate forest and pasture samples, respectively.

**FIGURE S2.** Relative abundances of bacterial (A) and archaeal (B) communities at phylum level in the metagenomes of soils under Amazon forests (green) and pastures (red). Taxa in each of the bacterial and archaeal domains represent over 98% of their taxonomic sequences. The most abundant phylum *Proteobacteria* is broken down into five classes. Error bars represent standard error of the mean (S.E.M). *P*‐value is calculated using Mann–Whitney test with 1000 permutations and symbols * and *** indicate significance values of *p* < 0.05 and *p* < 0.001, respectively.

**FIGURE S3.** Relative abundances of functional categories at KEGG level 2 in the soil metagenomes obtained from the Amazon forests (green) and pastures (red). Error bars represent standard error of the mean (S.E.M). Symbols * and *** indicate significance values of *p* < 0.05 and *p* < 0.001, which were calculated using Mann–Whitney test with 1000 permutations.

**FIGURE S4.** KEGG pathway map of differentially abundant genes observed in forest and pasture metagenomes for amino acid metabolism of (A) oxaloacetate acid, (B) pentose phosphate pathway, (C) pyruvate, and (D) alpha‐ketoglutarate. Only genes that passed selection criteria for both DESeq2 (BH‐adjusted *p* < 0.05) and Random Forest (importance score > 0.0001) methods are represented. Arrows indicate the enzyme‐mediated steps of the pathway, with a KEGG EC numeric classification representing the reaction being catalysed. A green arrow means that the abundance of a gene encoding a KEGG EC enzyme was higher in forests, while red means higher in pastures. Black arrows indicate genes with similar abundances between forest and pasture metagenomes.

**FIGURE S5.** KEGG pathway map for xenobiotic metabolism involved in benzoate, dioxin, and polycyclic aromatic hydrocarbon degradation. Only genes that passed selection criteria for both DESeq2 (BH‐adjusted *p* < 0.05) and Random Forest (importance score > 0.0001) methods are represented. Arrows indicate the enzyme‐mediated steps of the pathway, with a KEGG EC numeric classification representing the reaction being catalysed. A green arrow means that the abundance of a gene encoding a KEGG EC enzyme was higher in forests, while red means higher in pastures. Black arrows indicate genes with similar abundances between forest and pasture metagenomes.

**FIGURE S6.** Relationships of protein‐coding (KO) gene compositions with soil physicochemical factors. Vector fitted principal coordinates analysis of protein‐coding genes with vectors representing different soil factors. Each vector points to the direction of an increase in the gradient for the corresponding variable and length proportional to the correlation between ordination and variable. The circles (green, forest; red, pasture) represent the relative positions of protein‐coding genes observed in a sample. The significances (*P*‐values) of the vectors were calculated based on 999 random permutations of the data. Only soil physicochemical factors that were estimated to be significantly (*p* < 0.01) associated with PCo1 are shown. Bray–Curtis metric was used for estimating distances between different samples.

**FIGURE S7.** Relationships of viral composition with bacterial (A), archaeal (B), and protein‐coding gene (C) compositions across forest and pasture soil metagenomes. The relationships of compositional similarities are visualised using the principal coordinate loadings for the first axes (PCo1). The circles (green, forest; red, pasture) represent the relative positions of each soil taxonomic community. Bray–Curtis metric was used for estimating distances between different samples.

**TABLE S1.** Number of quality filtered sequence reads per soil sample used in this study.

**TABLE S2.** A complete list of bacterial (sheet A) and archaeal (sheet B) lineages at species level that discriminate soil metagenomes between forests and pastures. DESeq2 (BH‐adjusted *p* < 0.05) and Random Forest (importance score > 0.0001) methods were employed to calculate the discriminatory prokaryotic and viral species between forest and pasture soil metagenomes. Random Forest is a supervised classifier that classifies a subset of protein‐coding genes that are discriminatory between ecosystems. This classifier calculates an importance score for each feature (i.e., protein‐coding gene) by estimating the increase in generalised error when a feature is ignored. In our analysis, we considered 0.0001 as the cutoff for importance score. On the other hand, DESeq2 employs negative binomial distribution to identify the differentially abundant protein‐coding genes, where log_2_FC value is calculated to demonstrate the degree of variations in abundances for each protein‐coding gene. Here, the positive and negative log_2_FC values indicate the genes that are differentially abundant in forest and pasture metagenomes, respectively. The lfcSE stands for standard error of log_2_FC estimate. *P*‐value is calculated by the Wald statistic, which is the ratio of log_2_FC and its standard error. *P*‐values were adjusted for multiple comparisons using Benjamini‐Hochberg False Discovery Rate, and adjusted *p* < 0.05 was used for statistical significance.

**TABLE S3.** A complete list of protein‐coding genes at KEGG level 4 that discriminate soil metagenomes between forests and pastures. DESeq2 and Random Forest methods were employed to calculate the protein‐coding genes that differentiate between metagenomes from forest and pasture soils.

**TABLE S4.** A complete list of viral lineages at species level that discriminate soil metagenomes between forests and pastures. DESeq2 (BH‐adjusted *p* < 0.05) and Random Forest (importance score > 0.0001) methods were employed to calculate the discriminatory prokaryotic and viral species between soil metagenomes from forests and pastures.

**TABLE S5.** Soil physicochemical properties of the topsoil layer (0–10 cm) measured for samples collected from Amazon forests and pastures.

## Data Availability

Genetic data: Metagenomic sequences for all samples used in this study are available at the Joint Genome Institute IMG/M database under the following accession numbers: 1080879 to 1080888.
