# Supplementary figures and images for: Community‐Level Metabolic Shifts Following Land Use Change in the Amazon Rainforest Identified by a Supervised Machine Leaning Approach
Source: Environ Microbiol Rep. 2025 Apr 23;17(2):e70088. doi: 10.1111/1758-2229.70088 (PMC12018533; doi:10.1111/1758-2229.70088)

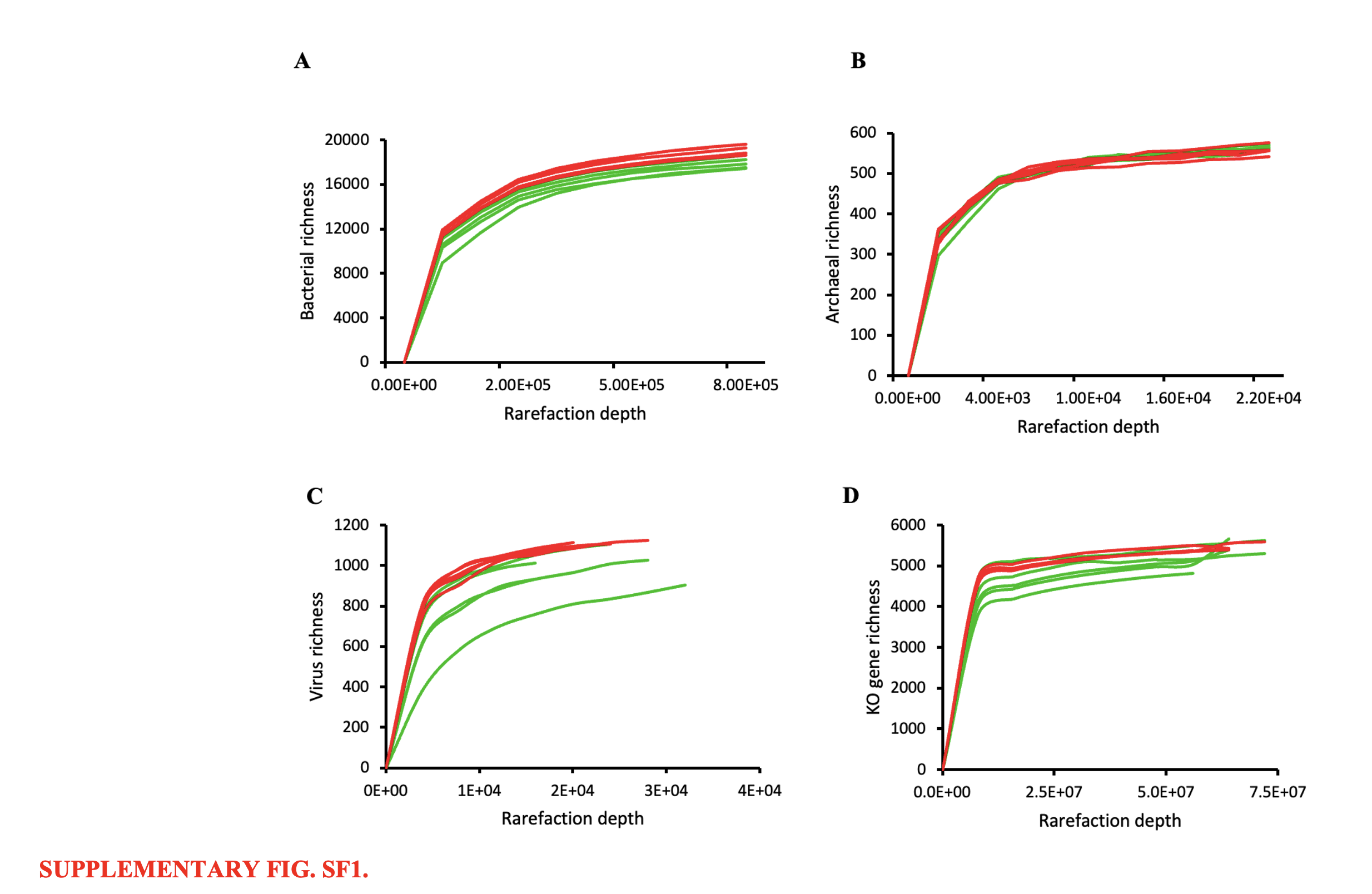

Supplement: Supplementary file 1 — FIGURE S1. Rarefaction curves of bacterial (A), archaeal (B), viral (C) taxonomic and protein‐coding (KO) (D) gene distributions of soil metagenomes obtained from Amazon forests and pastures. Species richness was used as a function of sequencing depth for forest and pasture samples. Green and red lines indicate forest and pasture samples, respectively. [file EMI4-17-e70088-s011.tif]

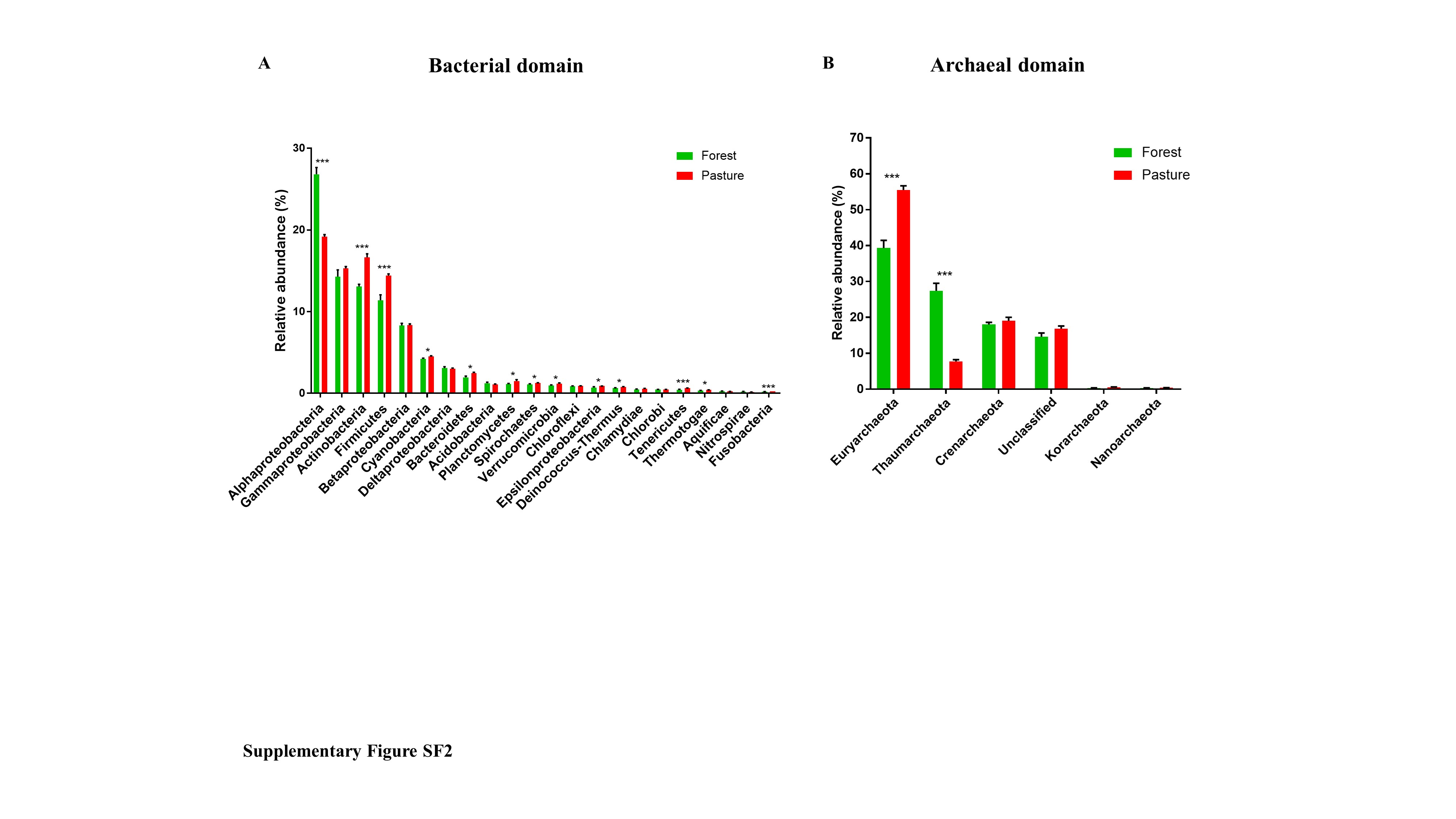

Supplement: Supplementary file 2 — FIGURE S2. Relative abundances of bacterial (A) and archaeal (B) communities at phylum level in the metagenomes of soils under Amazon forests (green) and pastures (red). Taxa in each of the bacterial and archaeal domains represent over 98% of their taxonomic sequences. The most abundant phylum Proteobacteria is broken down into five classes. Error bars represent standard error of the mean (S.E.M). P‐value is calculated using Mann–Whitney test with 1000 permutations and symbols * and *** indicate significance values of p < 0.05 and p < 0.001, respectively. [file EMI4-17-e70088-s008.tif]

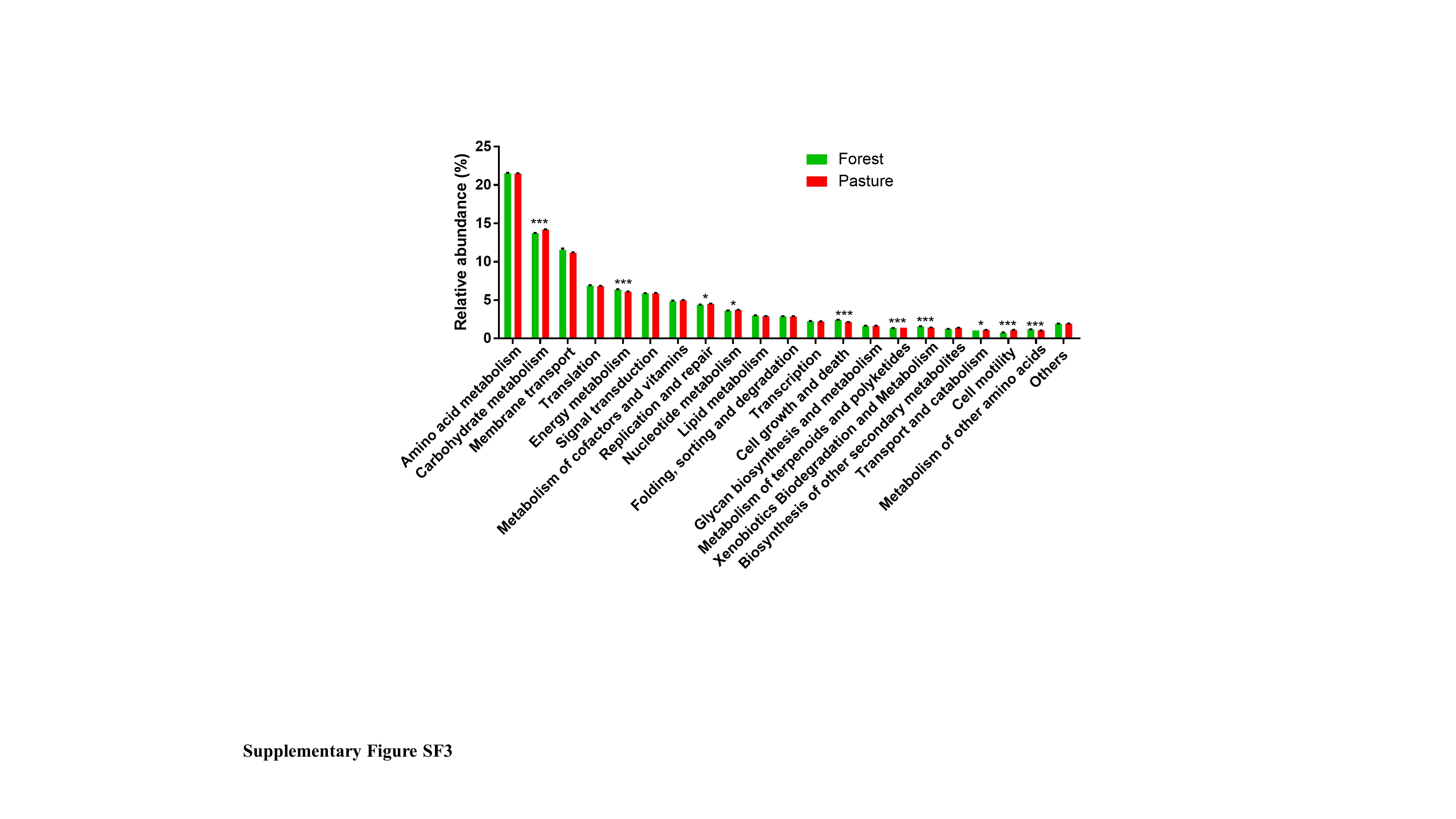

Supplement: Supplementary file 3 — FIGURE S3. Relative abundances of functional categories at KEGG level 2 in the soil metagenomes obtained from the Amazon forests (green) and pastures (red). Error bars represent standard error of the mean (S.E.M). Symbols * and *** indicate significance values of p < 0.05 and p < 0.001, which were calculated using Mann–Whitney test with 1000 permutations. [file EMI4-17-e70088-s012.tif]

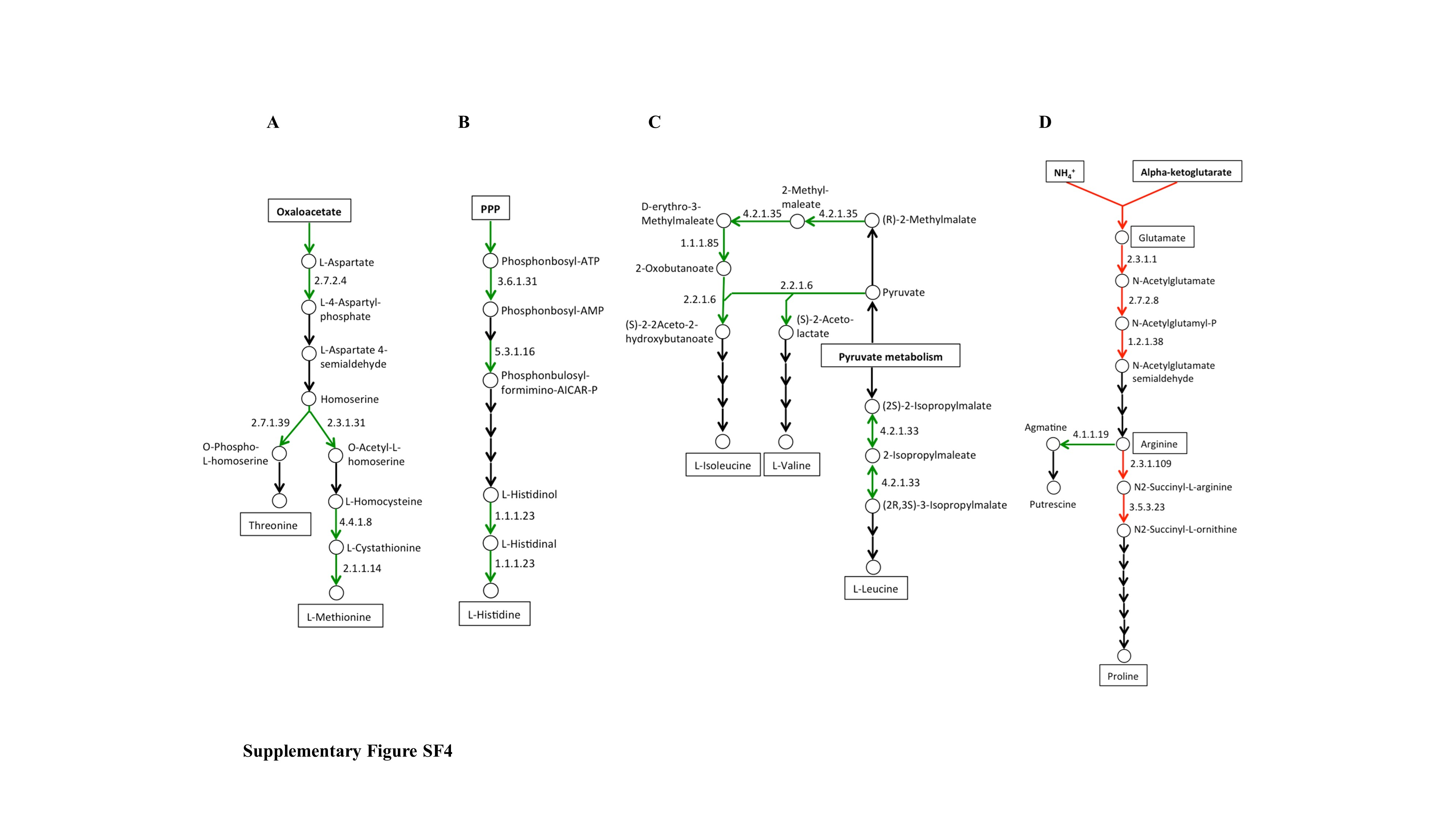

Supplement: Supplementary file 4 — FIGURE S4. KEGG pathway map of differentially abundant genes observed in forest and pasture metagenomes for amino acid metabolism of (A) oxaloacetate acid, (B) pentose phosphate pathway, (C) pyruvate, and (D) alpha‐ketoglutarate. Only genes that passed selection criteria for both DESeq2 (BH‐adjusted p < 0.05) and Random Forest (importance score > 0.0001) methods are represented. Arrows indicate the enzyme‐mediated steps of the pathway, with a KEGG EC numeric classification representing the reaction being catalysed. A green arrow means that the abundance of a gene encoding a KEGG EC enzyme was higher in forests, while red means higher in pastures. Black arrows indicate genes with similar abundances between forest and pasture metagenomes. [file EMI4-17-e70088-s002.tif]

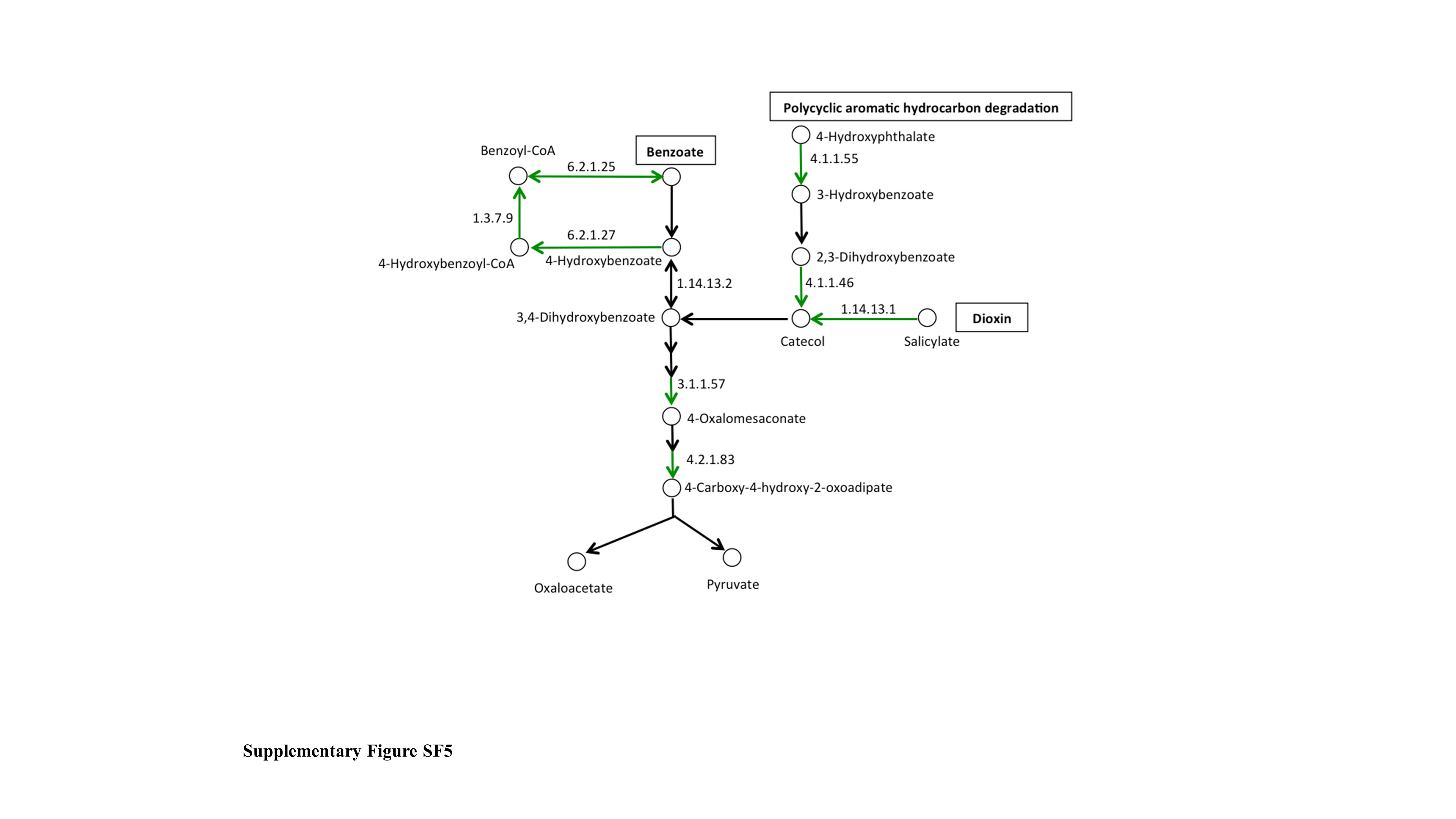

Supplement: Supplementary file 5 — FIGURE S5. KEGG pathway map for xenobiotic metabolism involved in benzoate, dioxin, and polycyclic aromatic hydrocarbon degradation. Only genes that passed selection criteria for both DESeq2 (BH‐adjusted p < 0.05) and Random Forest (importance score > 0.0001) methods are represented. Arrows indicate the enzyme‐mediated steps of the pathway, with a KEGG EC numeric classification representing the reaction being catalysed. A green arrow means that the abundance of a gene encoding a KEGG EC enzyme was higher in forests, while red means higher in pastures. Black arrows indicate genes with similar abundances between forest and pasture metagenomes. [file EMI4-17-e70088-s010.tif]

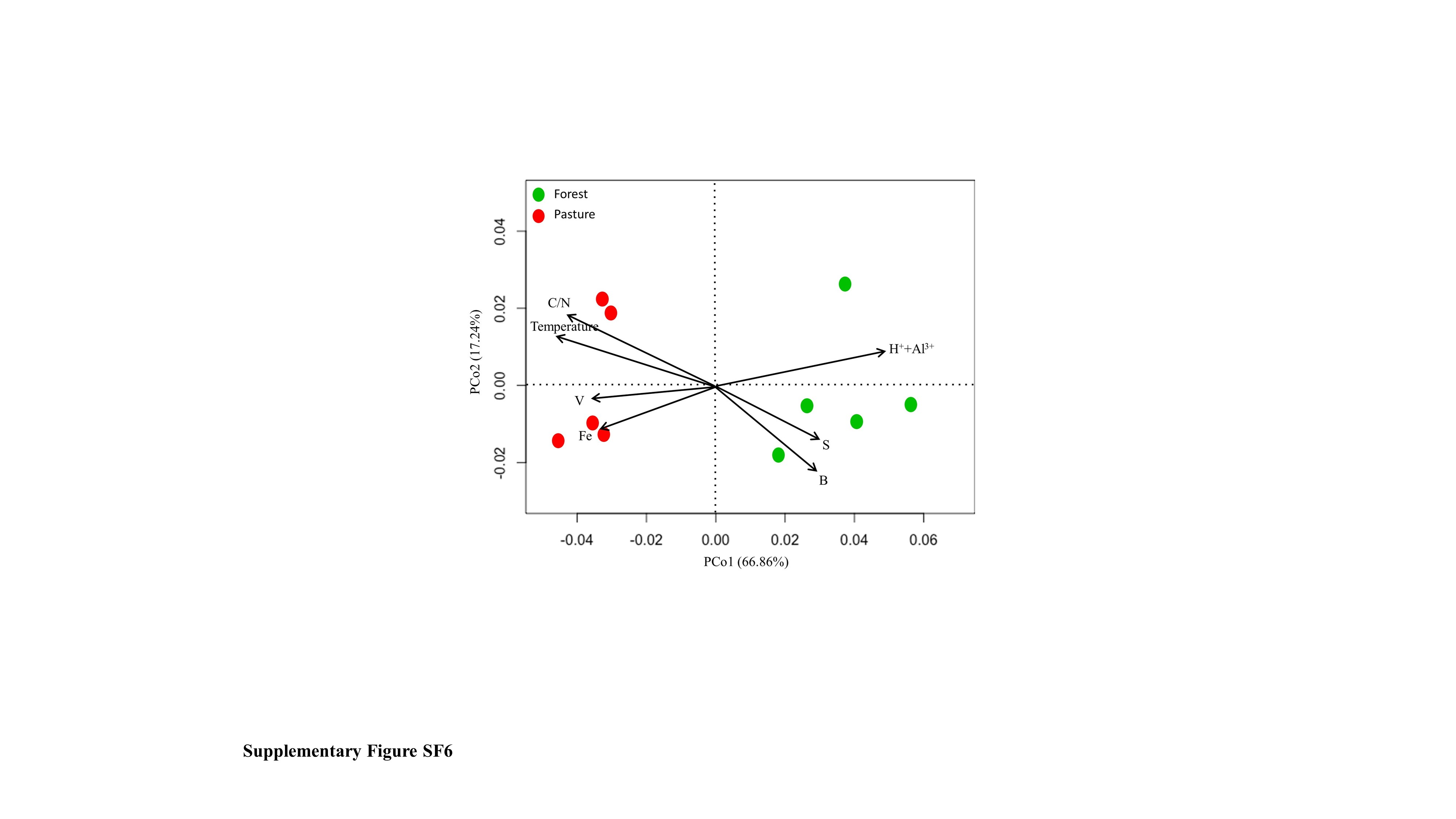

Supplement: Supplementary file 6 — FIGURE S6. Relationships of protein‐coding (KO) gene compositions with soil physicochemical factors. Vector fitted principal coordinates analysis of protein‐coding genes with vectors representing different soil factors. Each vector points to the direction of an increase in the gradient for the corresponding variable and length proportional to the correlation between ordination and variable. The circles (green, forest; red, pasture) represent the relative positions of protein‐coding genes observed in a sample. The significances (P‐values) of the vectors were calculated based on 999 random permutations of the data. Only soil physicochemical factors that were estimated to be significantly (p < 0.01) associated with PCo1 are shown. Bray–Curtis metric was used for estimating distances between different samples. [file EMI4-17-e70088-s005.tif]

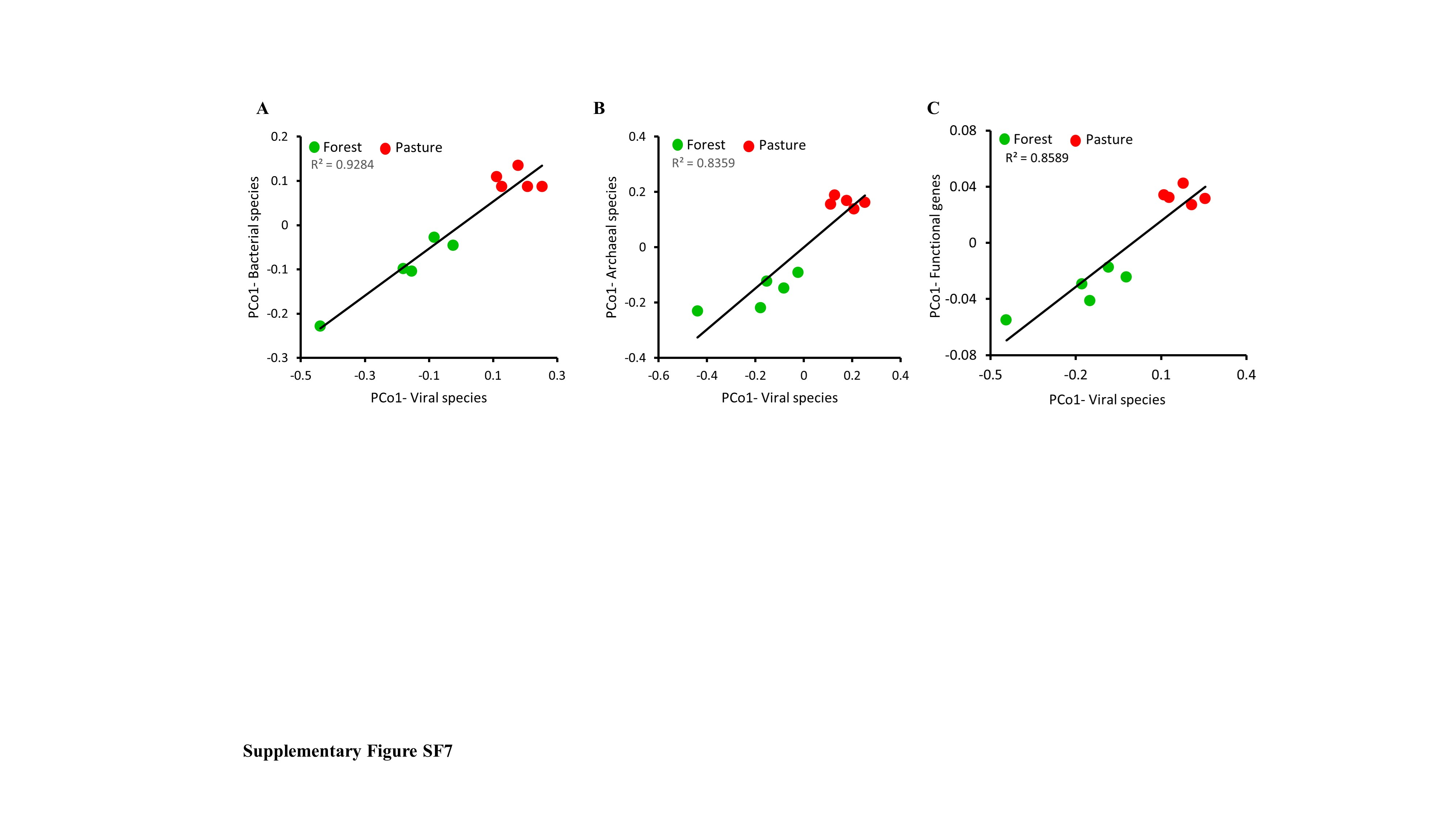

Supplement: Supplementary file 7 — FIGURE S7. Relationships of viral composition with bacterial (A), archaeal (B), and protein‐coding gene (C) compositions across forest and pasture soil metagenomes. The relationships of compositional similarities are visualised using the principal coordinate loadings for the first axes (PCo1). The circles (green, forest; red, pasture) represent the relative positions of each soil taxonomic community. Bray–Curtis metric was used for estimating distances between different samples. [file EMI4-17-e70088-s006.tif]
