# Supplementary material for: Community‐Level Metabolic Shifts Following Land Use Change in the Amazon Rainforest Identified by a Supervised Machine Leaning Approach
Source: Environ Microbiol Rep. 2025 Apr 23;17(2):e70088. doi: 10.1111/1758-2229.70088 (PMC12018533; doi:10.1111/1758-2229.70088)
Supplement: Supplementary file 12 — TABLE S5. Soil physicochemical properties of the topsoil layer (0–10 cm) measured for samples collected from Amazon forests and pastures. [file EMI4-17-e70088-s003.docx]

**Supplementary Table ST5.** Soil physicochemical properties of the top soil layer (0-10 cm) for the Amazon forest and pasture.

| **Soil Properties** | **Forest**  **(Average)^1^** | **Forest**  **(SEM)^2^** | **Pasture**  **(Average)^1^** | **Pasture**  **(SEM)^2^** | ***p*-value** | **Significance^3^** |
| --- | --- | --- | --- | --- | --- | --- |
| pH | 3.96 | 0.15 | 4.24 | 0.15 | 0.0317 | * |
| Temp (^o^C) | 24.86 | 0.11 | 27.14 | 0.16 | 0.0079 | ** |
| C | 1.117 | 0.01 | 1.498 | 0.2 | 0.0079 | ** |
| N | 0.0844 | 0.01 | 0.092 | 0.01 | 0.663 | ns |
| C/N | 13.456 | 0.69 | 17.116 | 0.49 | 0.0079 | ** |
| OM | 19.4 | 3.16 | 26.2 | 0.73 | 0.1429 | ns |
| P | 5.4 | 0.4 | 4.4 | 0.4 | 0.2778 | ns |
| K | 0.82 | 0.08 | 1.18 | 0.17 | 0.119 | ns |
| S | 9.6 | 0.4 | 8 | 0 | 0.0079 | ** |
| Ca | 10.2 | 1.11 | 10.4 | 1.4 | 0.4921 | ns |
| Mg | 4.2 | 1.46 | 2 | 0 | 0.0476 | * |
| Fe | 60.60 | 8.63 | 95.60 | 6.39 | 0.0238 | * |
| Mn | 53.44 | 10.15 | 29.08 | 3.52 | 0.0079 | ** |
| Zn | 0.88 | 0.06 | 1.28 | 0.17 | 0.0556 | ns |
| Cu | 1.12 | 0.19 | 1.24 | 0.16 | 0.2381 | ns |
| B | 0.206 | 0.01 | 0.164 | 0.01 | 0.0079 | ** |
| Al | 1.8 | 0.49 | 2 | 0 | 0.4048 | ns |
| H^+^ + Al^3+^ | 31 | 0 | 17.4 | 1.5 | 0.0079 | ** |
| m | 11.2 | 3.29 | 13.2 | 0.8 | 0.2302 | ns |
| SB | 15.22 | 2.6 | 13.58 | 1.23 | 0.4365 | ns |
| CEC | 46.22 | 2.6 | 30.98 | 2.58 | 0.0079 | ** |
| %V | 32.40 | 3.43 | 43.80 | 1.46 | 0.0397 | * |

Ca, Mg, K, Al, H^+^ + Al^3+^, SB, T are expressed in nmole.dm^-3^; P, S, Fe, Mn, Zn, Cu, B are expressed in mg.dm^-3^; OM = Organic Matter expressed as g.dm^-3^; m = Al saturation index, SB = Sum of bases; CEC = cation exchange capacity; and %V = Base saturation.

^1^ Values are averages based on five replicate points per site.

^2^ Standard Error of the Mean.

^3^ Tukey’s honestly significant different (HSD) test was performed between forest and pasture samples. Significant levels as: ns = *P* > 0.05, * = *P* < 0.05, ** = *P* < 0.01.
